# Alphaherpesvirus-induced activation of plasmacytoid dendritic cells depends on the viral glycoprotein gD and is inhibited by non-infectious light particles

**DOI:** 10.1371/journal.ppat.1010117

**Published:** 2021-11-29

**Authors:** Jonas L. Delva, Cliff Van Waesberghe, Barbara G. Klupp, Thomas C. Mettenleiter, Herman W. Favoreel

**Affiliations:** 1 Department of Virology, Parasitology, Immunology–Faculty of Veterinary Medicine–Ghent University, Merelbeke, Belgium; 2 Friedrich-Loeffler-Institut, Federal Research Institute for Animal Health, Greifswald-Insel Riems, Germany; Oklahoma State Univeristy, UNITED STATES

## Abstract

Plasmacytoid dendritic cells (pDC) are important innate immune cells during the onset of viral infections as they are specialized in the production of massive amounts of antiviral type I interferon (IFN). Alphaherpesviruses such as herpes simplex virus (HSV) or pseudorabies virus (PRV) are double stranded DNA viruses and potent stimulators of pDC. Detailed information on how PRV activates porcine pDC is lacking. Using PRV and porcine primary pDC, we report here that PRV virions, so-called heavy (H-)particles, trigger IFNα production by pDC, whereas light (L-) particles that lack viral DNA and capsid do not. Activation of pDC requires endosomal acidification and, importantly, depends on the PRV gD envelope glycoprotein and O-glycosylations. Intriguingly, both for PRV and HSV-1, we found that L-particles suppress H-particle-mediated activation of pDC, a process which again depends on viral gD. This is the first report describing that gD plays a critical role in alphaherpesvirus-induced pDC activation and that L-particles directly interfere with alphaherpesvirus-induced IFNα production by pDC.

## Author summary

The *Alphaherpesvirinae* subfamily of *Herpesviridae* encompasses several closely related viruses infecting humans and animals. They include herpes simplex virus 1 (HSV-1) causing cold sores and in severe cases encephalitis in man, and pseudorabies virus (PRV) causing respiratory and neurological symptoms in pigs. Alphaherpesviruses encode numerous immune evasive mechanisms that contribute to life-long infections of their host. Plasmacytoid dendritic cells (pDC) are specialized immune cells that are capable of producing massive amounts of antiviral type I interferons (IFN) upon virus infection. Here, we demonstrate that PRV-induced type I IFN production by pDC relies on the presence of complete virus particles and, importantly, that this activation depends on one specific viral protein, the envelope glycoprotein (g)D. We also found that noninfectious light (L)-particles, which lack nucleocapsids and are also produced during herpesvirus infection, interfere with both PRV- and HSV1-induced type I IFN production by pDC and that this again depends on gD. These data provide new insights on how alphaherpesviruses induce type I IFN responses in pDC and reveal that the enigmatic alphaherpesviruses L-particles counteract type I IFN production.

## Introduction

Alphaherpesviruses are enveloped double stranded DNA (dsDNA) viruses that are highly adapted to coexistence with their natural host, which is exemplified by their hallmark ability to establish life-long latent infections in the natural host. The alphaherpesvirus subfamily contains pathogens of humans and animals, including the human pathogens herpes simplex virus 1 and 2 (HSV1, HSV2) and varicella zoster virus (VZV), bovine herpes virus 1 (BoHV-1) in cattle, equine herpes virus 1 (EHV-1) in horses and suid herpes virus 1 (SuHV-1) or pseudorabies virus (PRV) in pigs [1]. PRV is the causative agent of Aujeszky’s disease causing respiratory, neurological and reproductive illnesses in pigs, and is often used as a model organism to study alphaherpesvirus-host interactions [2]. Curiously, alphaherpesvirus replication in host cells not only results in the formation of progeny infectious virions (so-called heavy or H-particles) but also in the generation of noninfectious light particles (L-particles). L-particles differ from H-particles in that they do not contain a nucleocapsid. All alphaherpesviruses tested thus far produce L-particles, i.e. HSV-1, PRV, EHV-1, BoHV-1 and VZV [3]. Deletion of viral genes that are required for capsid maturation, i.e. the UL25 gene that encodes a minor capsid protein, leads to exclusive L-particle production in infected cells [4,5]. Although L-particles are produced *in vivo* [6,7], information about their biological role is scarce. It has been suggested that they may act as immune decoys by capturing antibodies or may prepare uninfected cells for infection [7]. More recently, L-particles produced by HSV-1-infected monocyte derived dendritic cells (MoDC) were found to downregulate surface expression of the co-stimulatory protein CD83 and the IL-6 receptor on bystander MoDC [8,9].

Plasmacytoid dendritic cells (pDC) are a unique subset of leukocytes capable of producing immense amounts of type I IFNs. They were first described in humans in 1999 [10,11] as interferon producing cells (IPC) but were later also described in several other species, including mice [12], rats [13], monkeys [14], cattle [15], horses [16] and pigs [17]. pDC are sentinel cells that circulate between the blood and secondary lymphoid organs and are quickly translocated to sites of infection. pDC generally represent 0.1 to 0.5% of the peripheral mononuclear blood cell (PBMC) population, yet can produce up to 1,000 times more type I IFNs than any other cell type [18]. Type I IFNs and pDC are of particular importance in keeping alphaherpesvirus replication under control [19–24]. Importantly, pDC were identified as the major source of type I IFN within the PBMC population upon HSV stimulation [11]. In addition to their massive type I IFN production capacity, activated pDC also produce several other pro-inflammatory and Th1 cytokines upon stimulation and may present viral antigens to both CD4+ and CD8+ T-cells, thereby providing a powerful link between the innate and adaptive immune response [25–29]. Activation of pDC generally occurs through the endosomal nucleic acid toll like receptors (TLR)7 or TLR9 by pathogen derived single stranded RNA or double stranded unmethylated CpG-rich DNA, respectively [30]. Alphaherpesviruses like HSV and PRV potently stimulate type I IFN production by pDC [31–33]. Although HSV activation of pDC has been reported to occur via TLR9-mediated recognition of the viral genome [34,35], it has been suggested that alphaherpesviral envelope glycoproteins may directly and/or indirectly play a role in pDC activation [36].

Here, we report that viral DNA- and capsid containing PRV H-particles, but not L-particles, trigger IFNα production by primary porcine pDC. Importantly, we found that the viral gD envelope glycoprotein is required for efficient pDC activation. Moreover, we demonstrate that L-particles of both PRV and HSV-1 suppress H-particle-induced IFNα production by pDC, a process that again depends on gD.

## Materials and methods

### Ethics statement

Porcine blood was collected from 2–6 months old pigs that were housed at the Faculty of Veterinary Medicine of Ghent University. Housing and blood taking were approved by the Ethical Committee of the Faculty of Veterinary Medicine, Ghent University (approval EC2017/121).

### Cells and viruses

Swine testis (ST) cells were cultured in Earle’s minimum essential medium (MEM) with 10% fetal calf serum (FCS), 1 mM sodium pyruvate and antibiotics (100 U/ml penicillin, 0.1 mg/ml streptomycin, 0.05 mg/ml gentamycin) (Life Technologies). RK-13 cells were cultured in Dulbecco’s Modified Eagle’s Medium (DMEM) supplemented with 10% FCS and antibiotics. RK-13 cells stably expressing pUL25 [4], gB [37] or gD [38] were cultured with 0.5 mg/ml G418 (Life Technologies) instead of the aforementioned antibiotics, except when used in experimental assays. MDBK cells expressing gD [39] were cultured in DMEM with 10% FCS, 2 mM L-glutamine and 0.5 mg/ml G418. Vero cells expressing gH [40] or gL [41] were cultured in MEM with 10% FCS, 2 mM L-glutamine and 0.5 mg/ml G418. BHK-21 cells were cultured in DMEM with 5% FCS, 1 mM sodium pyruvate, 2 mM L-glutamine, 1 mM non-essential amino acids and antibiotics. PBMC and pDC were cultured in RPMI 1640 (Life Technologies) with 10% FCS, 2 mM L- glutamine, 1 mM sodium pyruvate, 1 mM non-essential amino acids, 20 μM ß-mercaptoethanol and antibiotics. Monocytes were cultured in pDC medium without ß-mercaptoethanol (monocyte medium).

All viruses used in the current study have been described before, including wild type (WT) PRV Kaplan strain [42] and isogenic strains deleted for a single gene locus, i.e. gBnull [37], gDnull [39], gEnull [43], gHnull [40], gLnull [41], gMnull [44] and UL25null [4]. The WT Becker strain [45] and the isogenic PRV151 strain, which contains a CMV-EGFP reporter gene inserted in the gG gene locus [46], were a kind gift of Dr. L. Enquist (Princeton University, USA). The HSV-1 KOS strain [47] was kindly provided by Dr. G. Campadelli-Fiume (University of Bologna, Italy). PRV stocks were titrated on ST monolayers or the respective complementing cell line and the HSV-1 strain was titrated on Vero monolayers.

### Antibodies and reagents

Antibodies against PRV glycoproteins gB (1C11, mIgG2a, non-neutralizing), gD (13D12, mIgG1, neutralizing) and gE (18E8, mIgG1, non-neutralizing) and irrelevant isotype control antibodies against bacterial FedF (Imm03, mIgG2a) and bacterial F4 (Imm01, mIgG1) have been described before [48–50] and were purified using HiTrap Protein GHP (GE Healthcare) according to the instructions of the manufacturer. Antibodies against VP5 (3C10) and US3 (8F86, mIgG1) [51] were kindly provided by Dr. L. Enquist (Princeton University, USA). HRP conjugated goat anti-mouse IgG was purchased from Dako (P0447). HRP labelled alpha-tubulin antibodies were obtained from Abcam (Ab40742). For magnetic activated cell sorting (MACS) and flowcytometric analyses, antibodies directed against CD4 (74-12-4, mIgG2b) [52] and CD172a (74-33-15, mIgG1) [52] were used and were a kind gift from Dr. A. Saalmüller (University of Vienna, AUT). Mouse anti-CD14 antibodies (MIL-2, mIgG2b) [53] were kindly provided by Dr. K. Haverson (Bristol University, UK) and antibodies against nectin-1 (CK6 (flow cytometry), CK41 (blocking assay) mIgG1) were kindly provided by Dr. C. Krummenacher (Rowan University, New Jersey) [54]. In some assays (when co-staining for nectin-1, using the CK6 antibody of the same mIgG1 isotype), the CD172a antibody was directly labelled using the DyLight 650 kit from Thermo Fisher (84535). PE labelled streptavidin (SA10041), AlexaFluor647 conjugated goat anti-mouse IgG1 (A21240), AlexaFluor488 conjugated goat anti-mouse IgG1 (A21121) and SytoxBlue live/dead marker (S34857) were purchased from Life Technologies. Propidium iodide was obtained from Invitrogen (P3566). MACS anti-mouse IgG microbeads and anti-mouse IgG1 microbeads were purchased from Miltenyi Biotech. For ELISA, the porcine IFNα antibodies F17 and K9 (both mIgG1) were kindly donated by Dr. B. Charley (INRA, France) [55]. PNGase F and the protein deglycosylation mix II were purchased from New England BioLabs (P6044 and P0704). 3,3’,5,5’-Tetramethylbenzidine one component substrate (TMB) was purchased from Bethyl Laboratories, recombinant porcine IFN-α from PBL Assay Science and streptavidin-HRP from Thermo Scientific. Antibodies against CD4 and IFNα (K9) were biotinylated using EZ-Link Sulfo-NHS-Biotin (Life Technologies) following the manufacturer’s instructions. Type A CpG oligonucleotide D32 [17] was synthesized by Integrated DNA Technologies. Pritelivir (BAY 57–1293) was purchased from Selleckchem (HY-15303), chloroquine from Tocris Bioscience (4109) and BSA (Fraction V) and Cytochalasin D from Sigma-Aldrich (resp. 1120180100 and C8273).

### Generation of phenotypically negative PRV virions

Supernatants containing virions that are both genotypically and phenotypically negative for a single glycoprotein or containing solely L-particles using UL25null PRV were generated as follows. First, mutant virions genotypically negative but phenotypically positive for a single glycoprotein or pUL25 were grown on corresponding complementing cell lines. These virions were then used to infect non-complementing ST cells at a multiplicity of infection (MOI) of 10 in MEM at 37°C for 2 h, after which the inoculum was removed by washing and the non-entered virions were inactivated by citrate treatment for 2min (40 mM sodium citrate, 10 mM KCl, 135 mM NaCl; pH 3). Afterwards, cells were washed 2 times with MEM, overlaid with pDC medium and collected at 24 h post inoculation (hpi). Cell debris was removed by centrifuging 10 min at 1,000 g. The absence of infectious virus was validated by titration on ST cells.

### Isolation of H- and L-particles

For PRV, H- and L-particles were purified as described before [56] with some minor adjustments. Briefly, 175 cm^2^ flasks with confluent ST cells were either mock-inoculated or inoculated with UL25null PRV Kaplan or WT PRV Kaplan at an MOI of 10 in MEM at 37°C. At 2 hpi, non-entered virions were inactivated by citrate treatment for 2 min (40 mM sodium citrate, 10 mM KCl, 135 mM NaCl; pH 3), after which the cells were washed two times and overlaid with ST medium. Supernatant was collected at 24hpi and cell debris was removed by centrifuging 10 min at 1,000 G and a 0.45 μm filtration step. Viral particles (or mock samples) were pelleted for 1 h at 20,000 G using a Type-35 rotor (Beckman Coulter), resuspended in 0.5 ml PBS, briefly sonicated and carefully layered onto a 30–10% iodixanol (Sigma) gradient and subsequently centrifuged for 2 h at 68,400 G in a SW41-Ti rotor (Beckman Coulter). H- and L-particle bands were collected, aliquoted and stored at -80°C until further use.

For HSV, 80–90% confluent BHK-21 cells were infected with HSV-1 KOS at an MOI of 0.01 [56,57]. Upon full cytopathic effect, supernatant was collected and H- and L-particles were purified identically as for PRV, with the exception of using a linear Ficoll 400 (Sigma) gradient for H- and L-particle separation (SW41Ti, 2 h, 26,000 G) and diluting the collected H- and L-particles in PBS followed by an additional ultracentrifugation step (SW41Ti, 2 h, 80,000 G), resuspending the viral pellets in PBS and storing until further use. Viral protein concentrations were determined using a Pierce BCA kit according to the manufacturer’s instructions (ThermoFisher).

### Generation of fixed RK-13 cells in suspension

Confluent monolayers of parental RK-13 cells or RK-13-cells expressing either PRV gB or gD were gently detached using Accutase according to the manufacturer’s instructions (BioLegend), washed two times in PBS and fixed using a 3% paraformaldehyde solution for 10 minutes at room temperature after which cells were washed five times in pDC medium and counted using an ACEA Novocyte flow cytometer. For IFNα assays, a final concentration of 250,000 cells /mL was added to PBMC prior to stimulation with density gradient purified PRV H-particles or CpG ODN.

### Deglycosylation assays

For PRV virions, 10 μg density gradient purified PRV H-particles were deglycosylated with PNGase F, which removes N-linked glycans, or a deglycosylation mixture removing both O- and N-linked glycans according to the manufacturer’s instructions for non-denaturing reaction conditions. For RK-13 cells, confluent monolayers of parental RK-13 cells or RK-13 cells expressing PRV gD were gently detached with Accutase (BioLegend) and washed two times in PBS with 1% BSA and 1 μM of the actin polymerization inhibitor cytochalasine D (incubation buffer) to prevent endocytosis of deglycosylated gD. Subsequently, 6.6x10^6^ cells/mL were incubated for 30 min at room temperature in incubation buffer with or without 5 μL deglycosylation mixture per 10^6^ cells followed by 1 h incubation at 37°C under mild agitation. Cells were subsequently washed two times in PBS, paraformaldehyde fixed and added to PBMC as mentioned in the previous paragraph.

### Interferon assays

5x10^6^ PBMC /mL or 160,000 FACS-purified porcine pDC /mL were coincubated with WT Kaplan PRV or HSV-1 density gradient purified H-particles at a final TCID_50_ of 10^6.8^, unless mentioned otherwise, or 10 μg/mL CpG ODN D32 at 37°C for 22 h after which cell supernatants were collected and IFNα titers were determined using ELISA (see below).

### Antibody treatment of PRV H- or L-particles

Purified H-particles were diluted in pDC medium and incubated for 1 h at 37°C with or without monoclonal antibodies and subsequently added to PMBC or FACS purified pDC. Purified L-particles were diluted in pDC medium and incubated for 1 h at 37°C with or without monoclonal antibodies. Next, L-particles were pelleted by centrifuging for 30 min at 20,000 G in a 5424R Eppendorf centrifuge and the supernatant was removed. After two washing steps (resuspending in PBS followed by pelleting as described before), particles were resuspended and added to PBMC or FACS purified pDC.

### PBMC isolation, monocyte isolation and pDC purification

Porcine PBMC were collected as described before [32]. Briefly, PBMC were isolated from whole blood using a lymphoprep density gradient (Alere Technologies). After lysis of red blood cells in Tris-buffered ammonium chloride buffer, PBMC were washed and resuspended in pDC medium and counted.

For pDC depletion assays, pDC were stained for CD4 and CD172a and gated as described in the flow cytometry section. Either non-pDC or all live cells were sorted on the BD FACS Melody (BD Biosciences).

Monocytes were isolated as described before [58] by cultivating 5x10^6^ PBMC /mL for 48 h at 37°C and subsequently, unadhered lymphocytic cells were removed by washing three times with RPMI and finally overlaid with monocyte medium.

For pDC purification, pDC were first enriched as described in [32]. Enriched pDC were subsequently stained for CD4 and CD172a and gated as mentioned in the flow cytometry section. pDC were purity-sorted using a BD FACS Melody (BD Biosciences). Post sort analyses showed >99% purity of the resulting pDC population.

For human PBMC, buffy coats from healthy donors were purchased from Red Cross Belgium. Analogously, PBMC were separated on lymphoprep [59], red blood cells were lysed, washed, resuspended in pDC medium and counted.

### Flow cytometry

Monocytes cultivated for 48 h, confluent ST cells or fresh purity sorted pDC were either mock inoculated or inoculated with WT PRV Becker or the isogenic GFP-expressing PRV Becker strain PRV151 at an MOI of 10 and harvested at 24 hpi. Next, cells were incubated for 30 min at 4°C with propidium iodide (1/1,000), washed two times and GFP expression was analyzed using an ACEA Novocyte flow cytometer. For PBMC populations, cells were incubated with primary antibodies for 30 min at 4°C, washed three times and incubated 30 min at 4°C with secondary antibodies and SytoxBlue (1/1000) and analysed by flow cytometry. When necessary (CK6 staining), secondary antibodies were blocked with 5% mouse serum. pDC were gated based on FSC, SSC and the CD4^high^ and CD712a^dim^ phenotype, as described before [33].

### SDS-PAGE and Western blotting

Cells were collected at 4°C, washed in TNE buffer (50 mM Tris, 150 mM NaCl, 1 mM EDTA, pH 6.8) and lysed for 1 h at 4°C in TNE lysis buffer (TNE, 10% NP-40 (Roche) and protease inhibitor cocktail (Sigma-Aldrich)). Nuclei were removed by spinning 10 min at 10,000 G [32]. Purified H- and L-particles were lysed and heated for 5 min at 95°C in SDS-PAGE loading buffer without ß-mercaptoethanol or bromophenol blue. Protein concentrations of the cell and viral lysates were determined using a Pierce BCA kit according to the manufacturer’s instructions (ThermoFisher) after which the cell lysates were mixed with SDS-PAGE loading buffer and heated for 5 min at 95°C. ß-mercaptoethanol was added to the viral lysates used for the Coomassie staining and Western blot against VP5 and tubulin. 20 μg cellular or 2 μg viral proteins were loaded and run on a polyacrylamide gel (10%) via SDS-PAGE and either blotted on a Hybond-P PVDF membrane (GE Healthcare) or directly visualised using Coomassie brilliant blue staining (ThermoFisher). Membranes were blocked in blocking buffer (PBS with 5% milk powder (Nestlé), 0.1% Tween-20 (Sigma-Aldrich)) for 1 h at room temperature. Next, blots were incubated with primary antibodies at 4°C overnight, washed, and subsequently incubated with HRP conjugated secondary antibodies (HRP conjugated, goat α-mouse; used 1/2000) for 1 h at room temperature. Primary and secondary antibodies were diluted in blocking buffer. Finally, blots were developed using chemiluminescence.

### ELISA

Porcine IFNα concentrations were measured by ELISA as described before [32]. Human IFNα concentrations were measured using a pan hIFNα ELISA (MabTech) according to the instructions of the manufacturer.

### Q-PCR

For PRV genome quantification, total DNA was extracted from PRV virions using the DNeasy mini kit (Qiagen). Q-PCR amplifications were carried out with SYBR Green PCR master mix (ThermoFisher) following the manufacturer’s instructions. Primers were targeted to the US3 gene: Forward 5′ GACGGGGGGTTTCCTGATTTA and Reverse 5′ GTATCTCATCAGCGGAAGGGC. Genome copy numbers were determined according to a 10-fold diluted standard curve of the US3 plasmid (pKG1) [60] ranging from 10^1^ copies /5μL to 10^9^ copies /5μL.

### Transmission electron microscopy

Infected cells were prepared for and analysed by TEM, using a JEOL JEM-1400 Plus transmission electron microscope (JEOL), as described before [61].

### Statistical analysis

Statistical analysis was performed using GraphPad Prism. Data were analyzed using the student’s t-test or repeated measures ANOVA at the 5% significance level. For the latter, *post hoc* comparisons between different conditions were performed by Tukey’s range test.

## Results

### DNA containing PRV virions trigger pDC activation via endosomal acidification

**Fig 1:**
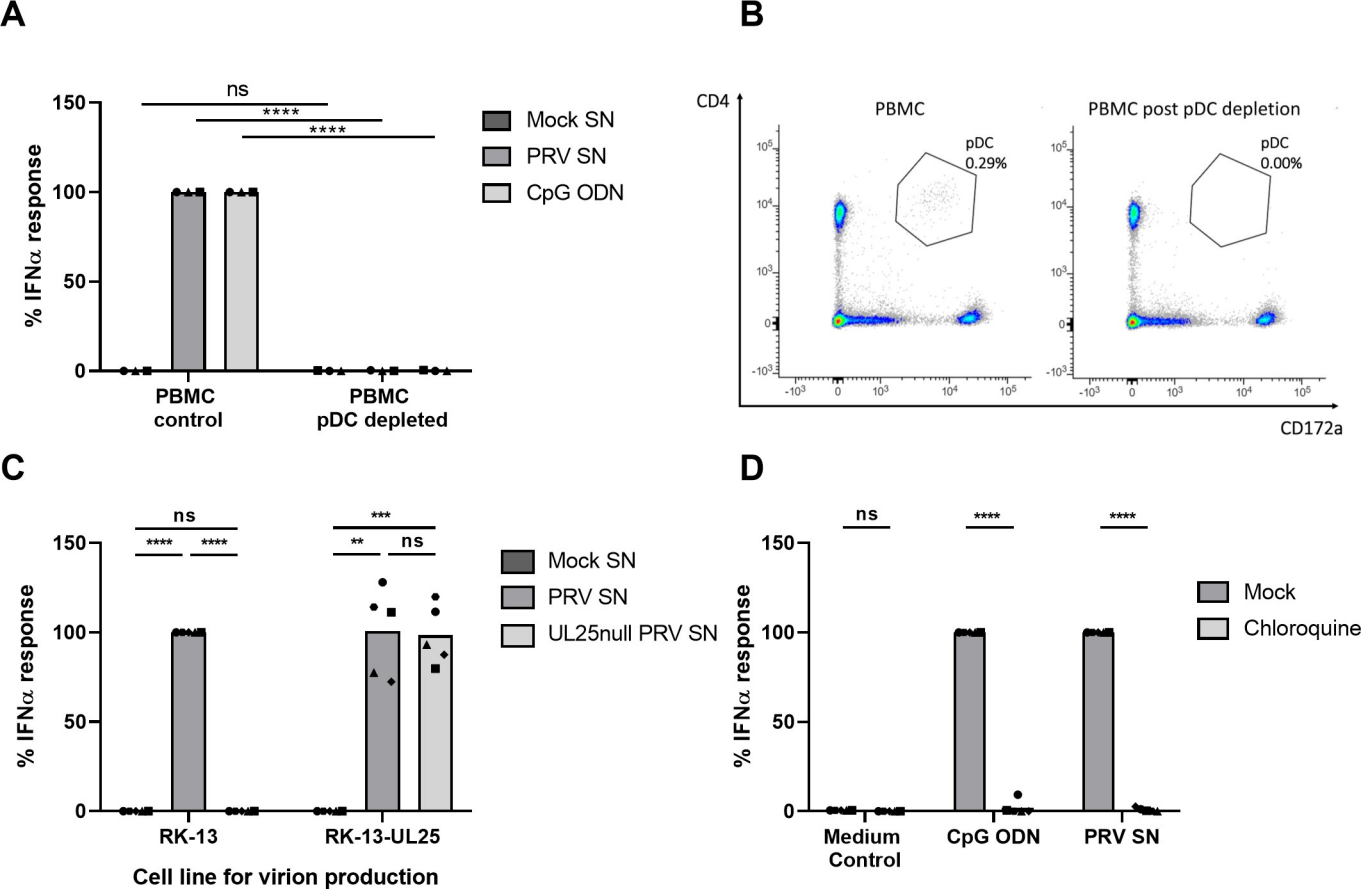
PRV-induced IFNα response by porcine PBMC depends on pDC, H-particles and endosomal acidification. (A) IFNα response of porcine PBMC either or not depleted for pDC upon stimulation with mock-, PRV supernatant (SN) or CpG ODN for 22h. PRV SN was collected from ST cells at 24hpi at a multiplicity of infection (MOI) of 10. IFNα levels were determined by ELISA. Data shown are normalized to the PBMC control response (set to 100) for each of the three independent repeats. (‘ns’ not significant, ‘****’ P<0.0001). (B) Post FACS sort analysis of porcine PBMC with either live cells sorted (left) or all cells except pDC sorted (right). pDC are characterized as CD4^high^CD172a^dim^ and indicated by the gate. (C) IFNα response upon porcine PBMC stimulation for 22h with supernatant collected at 24hpi from parental RK-13 cells or UL25-expressing RK-13 cells that were either mock infected or infected with WT PRV Kaplan or its isogenic UL25null mutant. Data shown are normalized to the response of PBMC stimulated with WT PRV produced on RK-13 cells (set to 100) for each of the five independent repeats. (‘ns’ not significant, ‘**’ P < 0.01, ‘***’ P < 0.001, ‘****’ P<0.0001). (D) Porcine PBMC were mock-treated or treated with 10μM chloroquine for 1h at 37°C followed by stimulation with CpG ODN, PRV SN or medium control for 22h. PRV SN was collected from ST cells at 24hpi at a multiplicity of infection (MOI) of 10. IFNα responses are depicted as normalized to the stimulus in the mock condition (set to 100) for each of the five independent repeats. (‘ns’ not significant, ‘****’ P<0.0001).

Earlier, we showed that PRV-infected cells trigger IFNα production by porcine pDC [32]. Here, we first investigated whether cell-free PRV is able to stimulate primary porcine pDC. Fig 1A shows that supernatant of PRV-infected cells triggers IFNα production by PBMC and that depletion of pDC from the PBMC population virtually abolishes IFNα production. The latter confirms that pDC represent the most important, if not the sole, source of IFNα production in the PBMC population in response to alphaherpesviruses, in line with earlier observations [11,32]. Efficiency of pDC depletion from the PBMC population is shown in Fig 1B.

Supernatant of PRV-infected cells not only contains mature PRV particles (so-called heavy or H-particles) but also light particles (L-particles) that lack DNA and capsid [62]. Although this has not yet been assessed in pDC, it has been suggested that alphaherpesvirus envelope glycoproteins, in the absence of viral DNA, may trigger type I IFN responses [63]. To investigate whether L-particles, which contain viral envelope glycoproteins but lack DNA, are able to trigger pDC activation, assays were performed using wild type (WT) PRV and an isogenic UL25null PRV strain. As the UL25 gene encodes for a minor capsid protein which is essential for newly produced viral nucleocapsids to leave the cell nucleus and assemble into mature progeny virions, deletion of this gene abolishes the production of DNA-containing virions in the supernatant while maintaining the capacity of the production of L-particles [4]. In contrast to supernatant derived from WT PRV-infected cells, supernatant derived from UL25null PRV-infected cells did not trigger an IFNα response (Fig 1C), whereas supernatant from UL25null PRV-infected cells that stably express the pUL25 gene product (RK-13-UL25), and therefore contains H-particles, did trigger IFNα production (Fig 1C). Together, these data indicate that DNA-containing virus particles are required for PRV-induced pDC activation, in line with the notion that the endosomal DNA sensor TLR9 is critical for pDC activation by HSV [34].

Next, we tested whether endosomal acidification is important for PRV-induced IFNα production by pDC. Fig 1D shows that chloroquine, an inhibitor of endosomal acidification, abolishes IFNα production by pDC in response to PRV and, as a control, in response to the TLR9 ligand CpG ODN D32. Hence, endosomal acidification is indeed required for PRV-induced activation of pDC.

### Efficient type I interferon production by porcine pDC does not require PRV replication but does depend on viral gD

**Fig 2:**
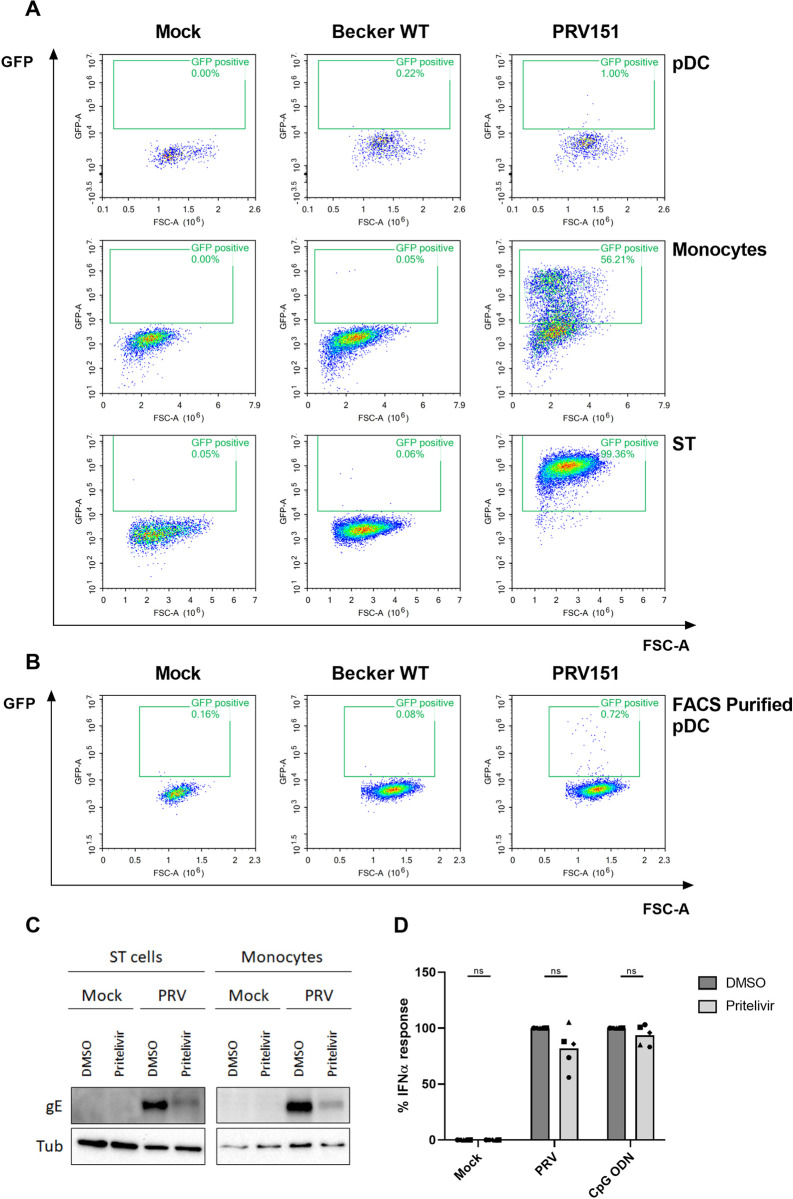
pDC are poorly susceptible to PRV infection and virus replication is not required for pDC activation. (A) Porcine PBMC, monocytes or ST cells were mock inoculated or inoculated with WT PRV Becker or the isogenic GFP-expressing PRV virus PRV151 (MOI 10) as mentioned in the materials and methods and 24h later analyzed for GFP expression by flow cytometry. pDC were gated from the PBMC population according to their CD4^high^CD172a^dim^ phenotype. One representative example out of four replicates is shown. (B) FACS purified porcine pDC were mock inoculated or inoculated with WT PRV Becker or the isogenic GFP-expressing PRV virus PRV151 (MOI 10) as mentioned in the materials and methods and 24h later analyzed for GFP expression by flow cytometry. (C) ST cells or monocytes were pre-treated with 10 μM pritelivir or DMSO control at 37°C for 30 min and subsequently mock-inoculated or infected with PRV (MOI 10). At 8 hpi, cells were collected and expression of the late viral protein gE and tubulin loading control were determined by Western blot. (D) Porcine PBMC were pre-treated with 10 μM pritelivir or DMSO control at 37°C for 30 min and subsequently mock-stimulated or stimulated with PRV density purified H-particles or CpG ODN for 22h. IFNα levels were determined by ELISA. IFNα responses are depicted as normalized to the stimulus in the control condition (set to 100) for each of the five independent repeats (‘ns’ not significant).

Our data indicate that DNA-containing PRV particles activate pDC via endosomal acidification. To assess whether pDC activation requires virus infection, as has been described for some viruses [64], we first investigated whether pDC are susceptible to PRV infection. Either virions of WT PRV strain Becker or an isogenic strain expressing soluble GFP under the control of the immediate early CMV promotor (PRV151) were added to PBMC. At 22hpi, cells were stained for different cell surface markers to discriminate different cell populations and analyzed by flow cytometry. As shown in Fig 2A, in samples inoculated with PRV151, virtually no GFP signal was detected in the pDC population, indicating that PRV does not or only very poorly infect pDC, in line with earlier reports [32]. As a positive control, substantial GFP expression was detected in monocytes or ST cells, which are known to be susceptible to PRV infection [58] (Fig 2A). Assays using FACS-purified pDC confirmed that pDC show a very limited susceptibility to PRV infection (<1% infected pDC at an MOI of 10, Fig 2B).

It is possible that PRV replication in non-pDC subpopulations of PBMC contributes to the observed PRV-induced pDC-mediated IFNα response. In addition, although our assays indicate only a very low susceptibility of pDC to PRV infection, this does not formally exclude that PRV replication in a small fraction of pDC may contribute to the observed IFNα response. Hence, to assess whether or not PRV replication in PBMC and/or pDC is involved in the observed PRV-induced IFNα response, assays were done in the presence of the viral helicase-primase complex inhibitor pritelivir (or DMSO diluent), which inhibits PRV DNA replication [65] and the subsequent expression of viral late proteins such as glycoprotein gE (Fig 2C). Addition of pritelivir did not affect the observed IFNα response, either upon stimulation of PBMC by PRV or by the TLR9 agonist CpG ODN (Fig 2D). Consequently, these results show that viral DNA replication or late gene expression in PBMC is not required to elicit a PRV particle-induced IFNα response by pDC.

**Fig 3:**
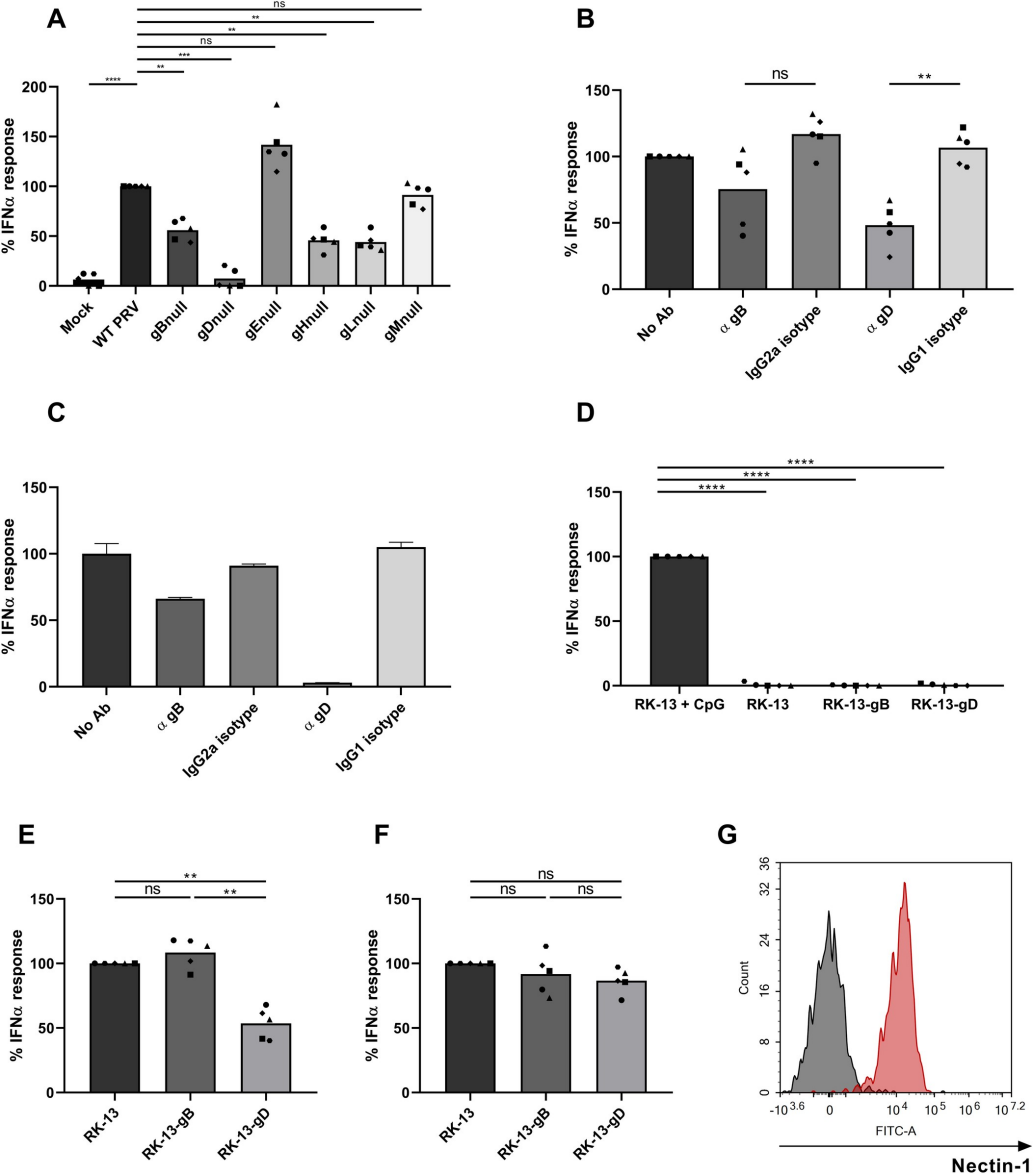
The PRV envelope glycoprotein gD is required for pDC activation. (A) Porcine PBMC were stimulated for 22h with supernatant from either mock-inoculated ST cells or ST cells infected with WT PRV Kaplan or isogenic single glycoprotein deletion mutants that was collected at 24hpi (and normalized based on viral genome levels). IFNα levels were determined by ELISA. IFNα responses are normalized to the WT PRV response (set to 100) for each of the five independent repeats. (‘ns’ not significant, ‘**’ P < 0.01, ‘***’ P < 0.001, ‘****’ P<0.0001). (B) PRV virions were incubated with or without different monoclonal antibodies (10 μg/mL) for 1h at 37°C and subsequently added to porcine PBMC for 22h. The measured IFNα responses are normalized to the condition without antibodies (set to 100) for each of the five independent repeats. (‘ns’ not significant, ‘**’ P < 0.01). (C) PRV virions were incubated with or without antibodies (10 μg/mL) for 1h at 37°C and subsequently added to FACS-purified porcine pDC for 22h (n = 1). The measured IFNα responses are normalized to the condition without antibodies (set to 100). Error bars represent the standard deviation of two technical replicates. (D) Porcine PBMC were added to parental RK-13 cells or RK-13 cells stably expressing either the PRV glycoprotein gB or gD for 22h. As a positive control, PBMC added to RK-13 cells were stimulated with CpG ODN. The measured IFNα responses are normalized to the condition including CpG ODN (set to 100) for each of the five independent repeats. (‘****’ P < 0.0001). (E-F) Parental RK-13 cells or RK-13 cells stably expressing either the PRV glycoproteins gB or gD were added to porcine PBMC prior to the addition of density gradient purified PRV H-particles (E) or CpG ODN (F) for 22h. The measured IFNα responses are normalized to the response when parental RK-13 cells were added (set to 100) for each condition for each of the five independent repeats. (‘ns’ not significant, ‘**’ P < 0.01). (G) Porcine PBMC were stained for CD4, CD172a and nectin-1 and analysed by flow cytometry. pDC were gated according to their CD4^high^CD172a^dim^ phenotype and nectin-1 expression (red) was measured compared to the isotype control (black).

Interaction of PRV virions with and subsequent infection of host cells occurs via specific interactions of viral envelope glycoproteins with the host cell surface. The sequence of events first consists of labile and rather unspecific interaction of viral gC with sugar moieties on the cell surface followed by a stable interaction between viral gD and specific cell surface receptors. Subsequently, additional virus glycoprotein-host factor interactions and activation of the gH/gL-gB machinery drive fusion of the envelope with the host membrane [66]. To assess whether the interaction between PRV and pDC that triggers an IFNα response relies on particular viral envelope glycoproteins or, alternatively, occurs via nonspecific interactions of pDC with virions, assays were performed using different mutant PRV strains deleted for specific viral envelope glycoproteins. Cell-free virions that were either WT or phenotypically negative for one of the viral envelope glycoproteins were used as stimulus. Since several viral envelope glycoproteins are essential for viral infectivity, virions that are phenotypically negative for these viral glycoproteins cannot be titrated. Hence, virus H-particles in supernatants were quantified by virtue of qPCR-based determination of viral genome copies and normalized before addition to pDC. Fig 3A shows that several of the tested deletion mutants show a substantial reduction in pDC activation. Deletion of the viral gM gene, which gene product is not involved in virus entry, did not affect the IFNα response, whereas deletion of the gE gene caused an increase in IFNα response, in line with our earlier identification of gE as a pDC-suppressive viral protein [32]. Whereas viruses devoid of gB, gH or gL, which all make part of the viral fusion complex involved in cell entry, all resulted in partially suppressed IFNα responses, virions that lacked gD failed almost completely in triggering an IFNα response by pDC. Hence, the viral fusion machinery appears to contribute to pDC activation, in line with data on HSV-1 [67], but gD appears to play a previously unknown and particularly important role in triggering a pDC-mediated IFNα response by PRV particles.

To confirm the importance of gD for activation of pDC by PRV, WT PRV virions were pre-incubated with monoclonal antibodies against gD or gB (or irrelevant isotype control antibodies) before addition to pDC. Fig 3B shows that the pDC-mediated IFNα response is markedly and significantly reduced when virus particles were pre-treated with a gD specific antibody, while pre-treatment with a gB-specific antibody did not lead to a statistically significant reduction in IFNα response. The inhibitory effect of the gD-specific antibody on PRV-induced IFNα production by pDC was even more dramatic when the assays were repeated using FACS-purified pDC (Fig 3C).

Although L-particles, which lack viral DNA or capsid but contain viral glycoproteins like gD, did not activate pDC (Fig 1C), we wanted to confirm that the gD glycoprotein by itself does not induce IFNα by pDC, as insect cells expressing HSV gD have been reported to trigger IFNα production by PBMC [63]. To this end, PBMC were added to either parental RK-13 cells or RK-13 cells stably expressing either PRV gD or PRV gB. RK-13 cells to which CpG was added were used as a positive control of IFNα production by PBMC. Neither parental RK-13 cells, gB-expressing RK-13 cells nor gD-expressing RK-13 cells triggered detectable IFNα production by PBMC (Fig 3D), implicating that the PRV glycoprotein gD on itself does not elicit detectable IFNα production by pDC.

Further in support of a critical role of the gD glycoprotein in PRV-induced IFNα production by pDC, addition of gD-expressing RK-13 cells, but not parental RK-13 cells or gB-expressing RK-13 cells, significantly suppressed the ability of PRV virions to trigger IFNα production by PBMC (Fig 3E), whereas none of the RK-13 cells (parental, gB-expressing or gD-expressing) suppressed CpG-triggered IFNα production by PBMC (Fig 3F).

Nectin-1 serves as the main gD receptor for several alphaherpesviruses and is expressed by human pDC [21,68]. Fig 3G demonstrates that porcine pDC also express nectin-1. However, when pDC were preincubated for 90min at 4°C with nectin-1-specific antibodies that interfere with the interaction between gD and nectin-1 [54], followed by addition of PRV and a switch to 37°C, no difference in IFNα production was observed, suggesting that nectin-1 does not contribute to the PRV gD-dependent pDC activation (S1 Fig).

### O-linked glycans on the viral gD glycoprotein are required for PRV induced pDC activation

**Fig 4:**
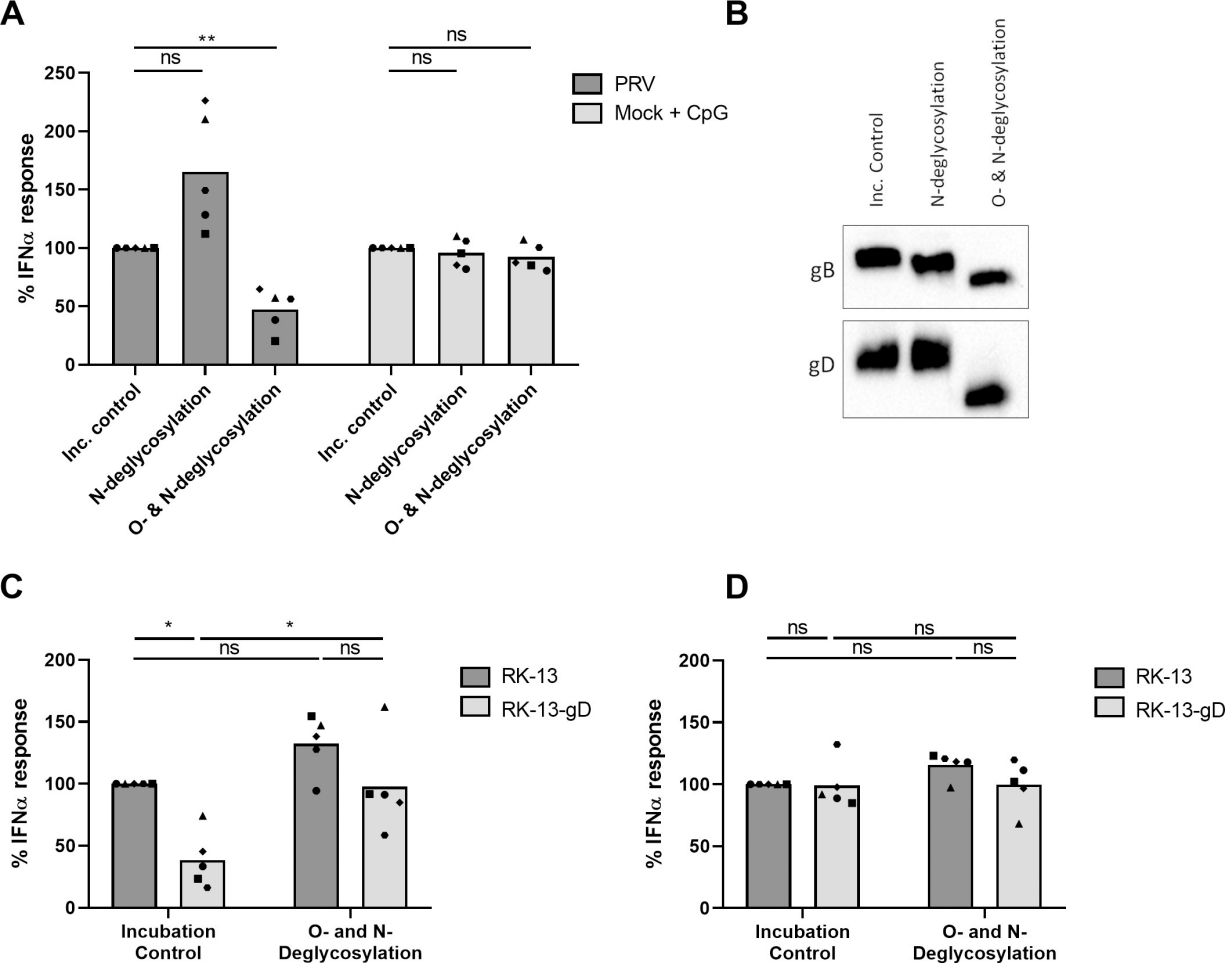
PRV gD-mediated activation of pDC depends on O-glycans. (A) Density gradient purified PRV H-particles or mock controls were incubated overnight at 37°C with PNGase F (removing N-glycans), a deglycosylation mixture (removing both N- and O-glycans) or deglycosylation buffer only as a control and subsequently added to porcine PBMC for 22h. For the mock control, pDC were stimulated with CpG ODN. The measured IFNα responses are normalized to the buffer control without any enzyme (set to 100) for each of the five independent repeats. (‘ns’ not significant, ‘**’ P < 0.01). (B) Western blot analysis of deglycosylated PRV H-particles. Antibodies against the viral proteins gB and gD were used. (C-D) Parental RK-13 cells or RK-13 cells stably expressing the PRV glycoprotein gD were incubated with a deglycosylation mixture (removing both N- and O-glycans) or PBS with 1% BSA only as an incubation control. Cells were subsequently added to porcine PBMC prior to the addition of density gradient purified PRV H-particles (C) or CpG ODN (D) for 22h. The measured IFNα responses are normalized to the response using incubation control parental RK-13 cells (set to 100) for each of the five independent repeats. (‘ns’ not significant, ‘*’ P < 0.05).

Given that several pDC receptors are glycan-binding lectins and that PRV gD is a glycoprotein that is O-glycosylated, but not N-glycosylated [69], we next wanted to investigate whether pDC activation by PRV relies on (O-)glycan structures. Therefore, virions were treated with glycosidases that remove either both O- and N-linked glycans or only N-linked glycans before addition to PBMC. A condition using CpG as pDC stimulus was used to rule out glycosidase-unrelated treatment effects. Removal of both O- and N-glycans from PRV virions significantly reduced IFNα production, whereas only removing N-glycans did not suppress (but rather increase) the PRV-induced IFNα response (Fig 4A). To check for successful glycosidase activity, treated virions were subjected to Western blot analysis. Fig 4B shows that, as expected, the apparent molecular mass of the N- and O-glycosylated gB was reduced by removing only N-glycans and reduced more dramatically by removing both O- and N-glycans. In addition, again in line with expectations [69], the apparent molecular mass of gD was only reduced when O-glycans were removed.

Given our previous results showing that addition of gD-expressing RK-13 cells interferes with PRV induced pDC activation, we investigated whether this effect could be reversed by removal of the O-linked glycans on gD. Hence, parental RK-13 or RK-13-gD cells were detached and pre-incubated with or without a glycosidase mixture, fixed and added in equal amounts to PBMC prior to PRV or CpG stimulation. Fig 4C demonstrates that glycosidase treatment indeed significantly suppressed the ability of RK-13-gD cells to reduce PRV-induced IFNα production by PBMC. In contrast, removal of the glycan structures on RK13 cells had no obvious effect on the CpG-induced IFNα production by PBMC (Fig 4D).

### L-particles inhibit the PRV-induced IFNα response by pDC

**Fig 5:**
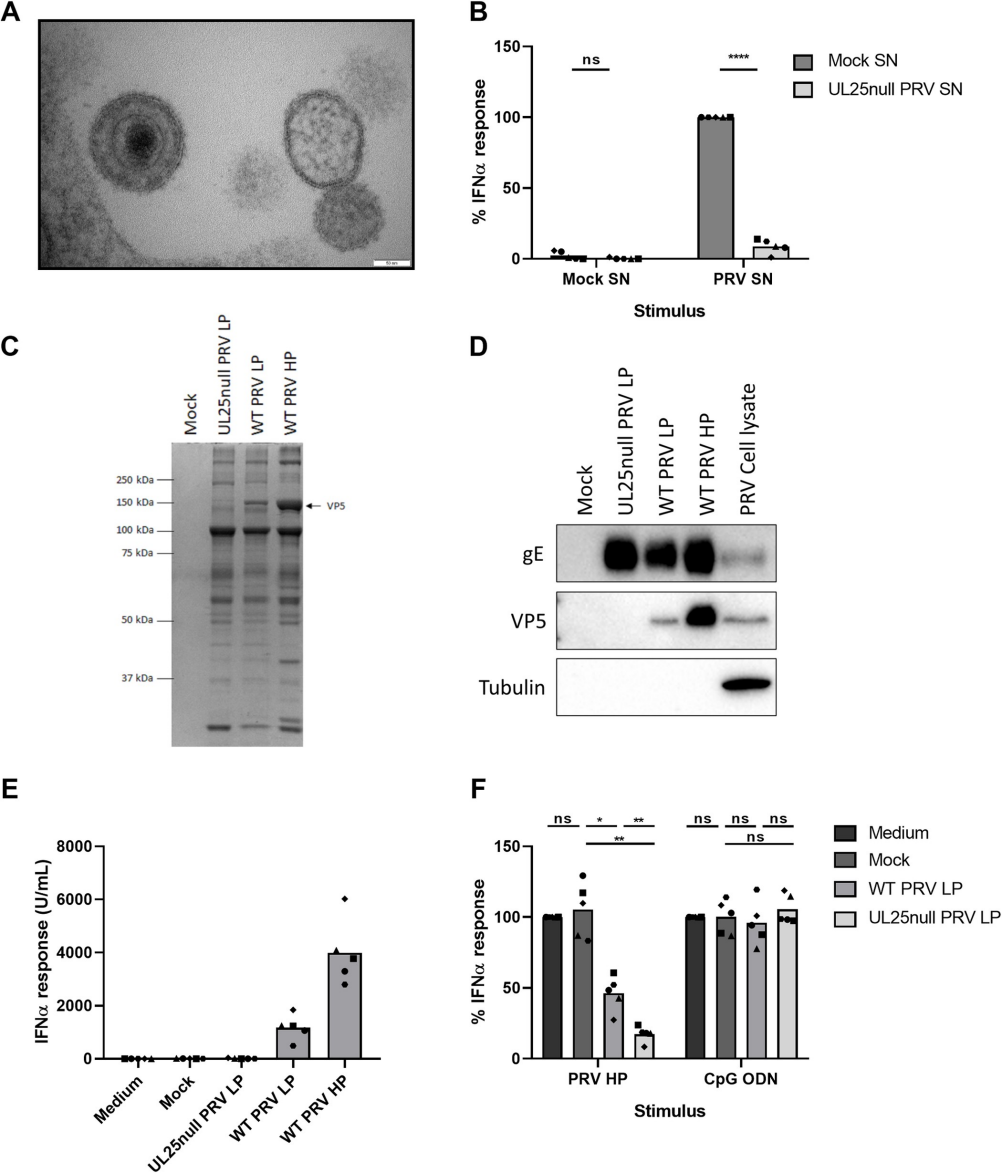
PRV L-particles inhibit the PRV H-particle-induced IFNα response by pDC. (A) Transmission electron microscopic picture of an L-particle (right) next to an H-particle (left) in a sample of PRV-infected ST cells at 12hpi. Scale bar represents 50 nm. (B) Porcine PBMC were stimulated for 22h with a combination of ten-fold diluted supernatant from mock-inoculated ST cells or WT PRV Kaplan-infected ST cells (MOI 10) collected at 24hpi and undiluted supernatant from mock-inoculated ST cells or UL25null PRV Kaplan-infected cells. The latter contains only L-particles and no H-particles. IFNα levels were determined by ELISA. IFNα responses are depicted as normalized to the response of PBMC stimulated with the combination of supernatant of PRV-infected cells and of mock-inoculated cells (set to 100) for each of the five independent repeats. (‘ns’ not significant, ‘****’ P<0.0001). (C) Coomassie blue staining of density gradient purified PRV L-particles from UL25null PRV Kaplan-infected ST cells and WT PRV Kaplan-infected ST cells and H-particles from WT PRV Kaplan-infected ST cells. Arrow indicates position of the VP5 major capsid protein that is present in H-particles but absent from L-particles. (D) Western blot analysis of density gradient purified PRV L-particles from UL25null PRV Kaplan-infected ST cells and WT PRV Kaplan-infected ST cells and H-particles from WT PRV Kaplan-infected ST cells. Antibodies against gE (envelope glycoprotein) and VP5 (capsid protein) and cellular protein tubulin were used. (E) Porcine PBMC were incubated with purified H- or L-particles, mock-treated or treated with medium control for 22h. Total IFNα production is shown of five independent repeats. (F) Porcine PBMC were coincubated with density gradient purified Kaplan PRV H-particles or CpG ODN and medium only, mock-purified sample, purified WT PRV L-particles or UL25null PRV L-particles for 22h. IFNα responses are normalized to the medium condition (set to 100) for each of the five independent repeats. (‘ns’ not significant, ‘*’ P < 0.05, ‘**’ P < 0.01).

Our findings indicate that an efficient PRV-induced IFNα response by pDC is driven by the viral gD glycoprotein. As mentioned before, alphaherpesvirus-infected cells not only produce infectious virions (H-particles) but also L-particles that are devoid of DNA and capsid (Fig 5A) [3]. Since L-particles contain the same set of viral glycoproteins as H-particles [56], but lack capsid and genome, we hypothesized that the presence of PRV L-particles during the interaction of PRV H-particles with pDC may interfere with efficient pDC activation. To examine this, supernatant from UL25null PRV-infected ST cells, containing only L-particles but no H-particles, was added to PBMC together with PRV H-particle-containing supernatant from WT PRV-infected cells in a 10 to 1 ratio. Fig 5B shows that the addition of supernatant from UL25null PRV-infected cells almost completely abrogates the IFNα response, suggesting that L-particles inhibit the pDC IFNα response induced by DNA-containing H-particles.

To exclude that, instead of L-particles, additional factors in the supernatant of UL25null PRV-infected cells (e.g. cytokines) suppress the PRV H-particle-induced IFNα response by pDC, and to assess whether L-particles derived from WT PRV also suppress the IFNα response, additional assays were performed using H- and L-particles of WT PRV and L-particles of UL25null PRV purified by density ultracentrifugation. As a control, a mock condition for L-particles was generated by subjecting supernatant from mock-infected cells to the same L-particle purification protocol. Purity of H- and L-particle fractions was confirmed by Coomassie staining and Western Blot analysis. Both the Coomassie gel and Western blot (Fig 5C and 5D) show that, as expected, the major capsid protein VP5 is prominently present in the H-particle lysate but is not detectable in the lysate of the UL25null L-particles. The L-particle fraction of the WT PRV sample, however, displays a weak but noticeable VP5 band, indicating that the WT PRV L-particle fraction contains some contaminating H-particles. As a control, cellular tubulin was only detected in the control lysate, but not in any of the viral preparations (Fig 5D).

Next, purified H- and L-particle fractions were checked for their capacity to stimulate IFNα production by pDC. Normalization was carried out based on total amount of protein before addition to PBMC. In line with our earlier observations, the pure L-particle fraction from UL25null PRV-infected cells did not trigger detectable IFNα (Fig 5E). In line with the slight H-particle contamination in the L-particle fraction from WT PRV-infected cells, a minor IFNα response was observed using this fraction.

To confirm that PRV L-particles indeed inhibit the H-particle-induced IFNα pDC response, purified L-particles were added in a 10:1 ratio to H-particles. As a control to assess whether L-particles may generally suppress pDC activity, L-particles were added to the TLR9 agonist CpG ODN D32. As shown in Fig 5F, addition of L-particles resulted in a drastic reduction in IFNα response triggered by H-particles but did not affect the CpG-induced IFNα response, confirming that PRV L-particles specifically suppress H-particle induced pDC activation. Moreover, when the highly pure UL25null L-particles were used instead of the L-particle fraction of WT PRV-infected cells, IFNα responses were almost entirely abolished, indicating that the slight contamination of H-particles in the WT PRV L-particle fraction counteracts to some extent the L-particle-mediated inhibition of pDC. Therefore, subsequent experiments were performed using the highly pure UL25null L-particle fraction.

### PRV L-particle-mediated inhibition of PRV-induced pDC activation is direct and dose dependent

**Fig 6:**
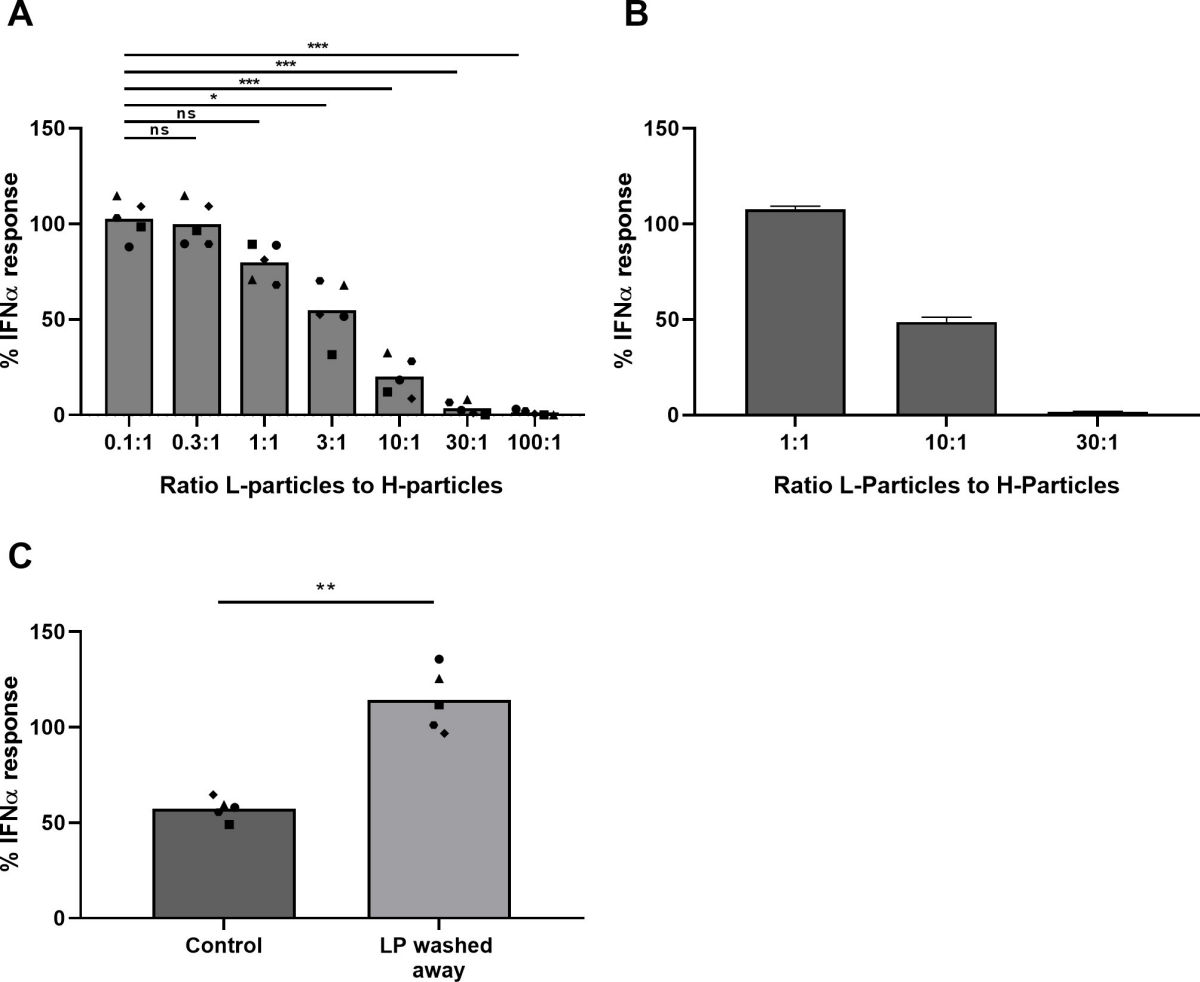
The L-particle mediated inhibition of the PRV H-particle-induced IFNα response by pDC is dose dependent. (A) Porcine PBMC were incubated for 22h with density gradient purified H-particles and different amounts of UL25null L-particles or a corresponding mock sample. IFNα levels were determined by ELISA. IFNα responses are normalized to the mock condition (set to 100) for each of the five independent repeats. (‘ns’ not significant, ‘*’ P < 0.05, ‘***’ P < 0.001). (B) FACS-purified porcine pDC were incubated for 22h with density gradient purified H-particles and different amounts of UL25null PRV L-particles or a corresponding mock sample (n = 1). The measured IFNα responses are normalized to the mock condition (set to 100) and error bars represent the standard deviation of two technical replicates. (C) Porcine PBMC were pre-incubated density purified UL25null L-particles or a corresponding mock sample for 2h at 37°C, washed and subsequently medium containing H-particles with or without the same number of L-particles as during the pre-incubation (corresponding to a 5:1 ratio of L-to-H-particles) was added. Following 22h of incubation at 37°C, supernatant was collected and IFNα concentrations were determined. IFNα responses are normalized to the mock condition (set to 100) for each of the five independent repeats. (‘**’ P < 0.01).

To assess whether L-particle-mediated inhibition of the PRV-induced IFNα response by pDC is dose dependent, different ratios of L- to H-particles were tested. Fig 6A shows that increasing doses of L-particles increasingly suppress H-particle-induced pDC activation. A ratio of L- to H-particles exceeding 10 completely abrogates the IFNα response.

To confirm that the observed results are not caused by secondary interactions between L-particles and other cell types in the PBMC population, assays were performed using FACS-purified pDC. Again, PRV H-particle-induced IFNα responses by purified pDC were inhibited by L-particles in a dose dependent manner (Fig 6B).

To determine whether pre-incubation of pDC with L-particles is sufficient to inhibit PRV-induced pDC activation, PBMC were pre-incubated with L-particles for 2 hours at 37°C followed by washing and addition of medium containing H-particles either or not supplemented with the same number of L-particles as during pre-incubation. IFNα responses were measured 22 hours later. Fig 6C shows that removal of pre-incubated L-particles before H-particle addition interfered with inhibition of the H-particle-induced IFNα response, implying that L-particles need to be present when H-particles interact with pDC to exert a suppressive effect.

### gD is involved in L-particle-mediated inhibition of PRV-induced pDC activation

**Fig 7:**
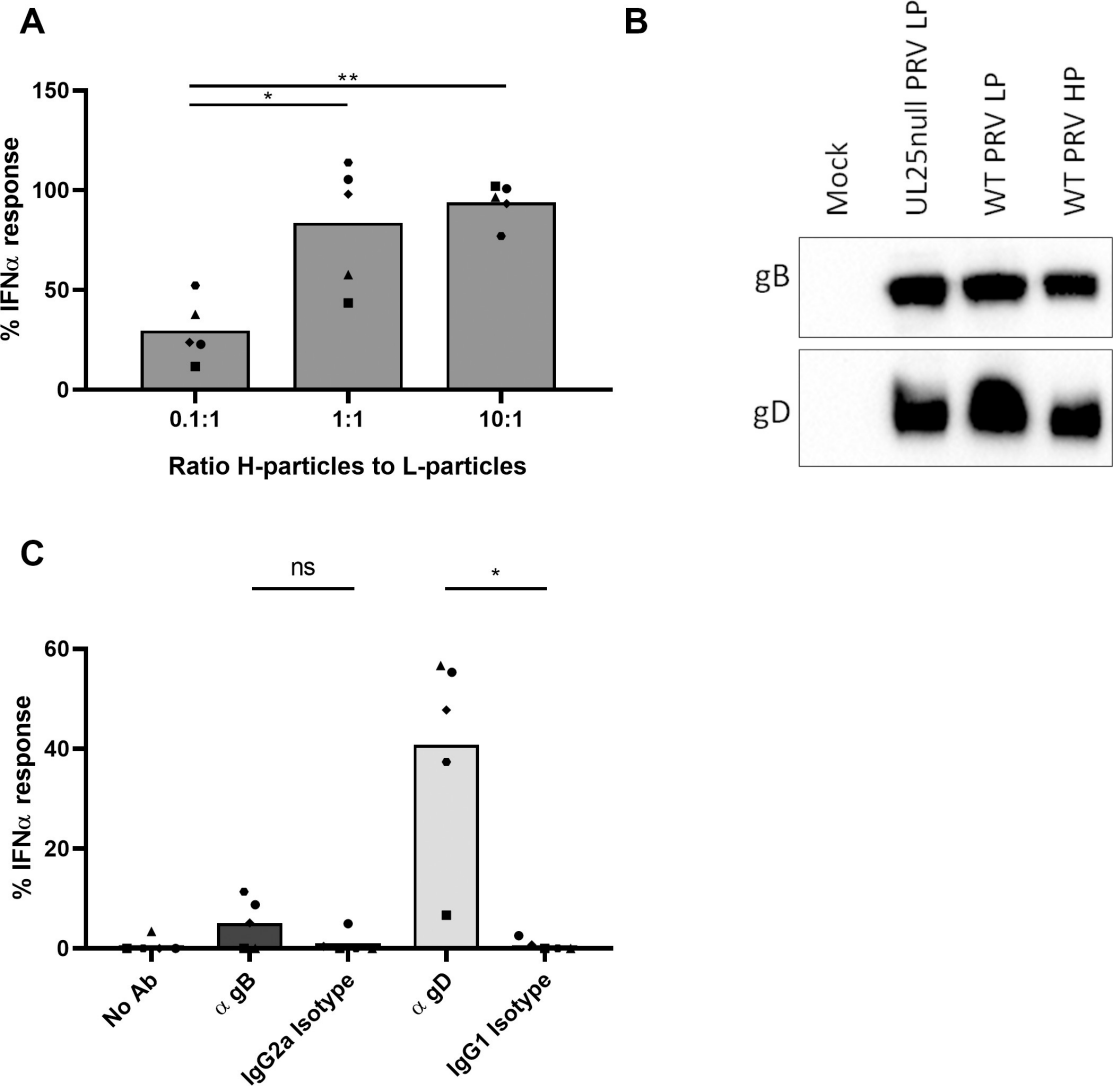
The inhibitory effect of PRV L-particles on H-particle-induced IFNα production by pDC depends on the viral gD envelope glycoprotein. (A) Porcine PBMC were incubated for 22h with a fixed number of L-particles or a corresponding mock sample and increasing numbers of H-particles. The number of H-particles in the lowest H-to-L-particle ratio of 0.1:1 corresponds to the same amount of H-particles as used in the previous experiments i.e. 10^6.8^ PFU/ml, with the two 10 fold increments of H-particles referred to as 1:1 and 10:1. IFNα responses are normalized to the mock condition (set to 100) for each of the five independent repeats. (‘ns’ not significant, ‘*’ P < 0.05, ‘**’ P < 0.01). (B) Western blot analysis of density gradient purified PRV L-particles from UL25null PRV Kaplan-infected ST cells and WT PRV Kaplan-infected ST cells and H-particles from WT PRV Kaplan-infected ST cells. Antibodies against the viral glycoproteins gB and gD were used. (C) Purified UL25null L-particles or corresponding mock samples were incubated with or without 10μg/mL of different monoclonal antibodies at 37°C for 1h, followed by two centrifugation steps and finally L-particles were resuspended in pDC medium and added to porcine PBMC together with H-particles (corresponding to a 100:1 ratio of L-to-H-particles). Supernatant was collected 22h later and IFNα responses are shown relative to the mock samples (set to 100) for each of the five independent repeats. (‘ns’ not significant, ‘*’ P < 0.05).

Our data are compatible with a model in which L-particles suppress H-particle-induced pDC activation via competitive inhibition. In line with this, Fig 7A shows that the addition of an increasing amount of H-particles to a constant number of L-particles overcomes L-particle-mediated inhibition of pDC. This suggests that H-particles and L-particles competitively bind to certain pDC receptors, where binding of sufficient H-particles leads to pDC activation.

Since we showed that PRV H-particles require gD to efficiently activate pDC, we wondered whether gD contributes to L-particle mediated inhibition of PRV-induced pDC activation. First, we confirmed that PRV L-particles incorporate at least similar amounts, if not more, of gD compared to H-particles (Fig 7B). Next, L-particles were pre-incubated with antibodies against gB or gD or the respective isotype control. Antibodies in the media were subsequently removed by pelleting and washing of the L-particles. Interestingly, pre-incubation of L-particles with a gD-specific antibody substantially and significantly reduced the inhibitory effect of L-particles on the H-particle elicited IFNα pDC response, while this was not the case using a gB-specific antibody (Fig 7C). These data suggest that L-particles compete with H-particles for gD-mediated interaction with and subsequent activation of pDC.

### L-particles from HSV-1 also suppress the H-particle-induced IFNα response

**Fig 8:**
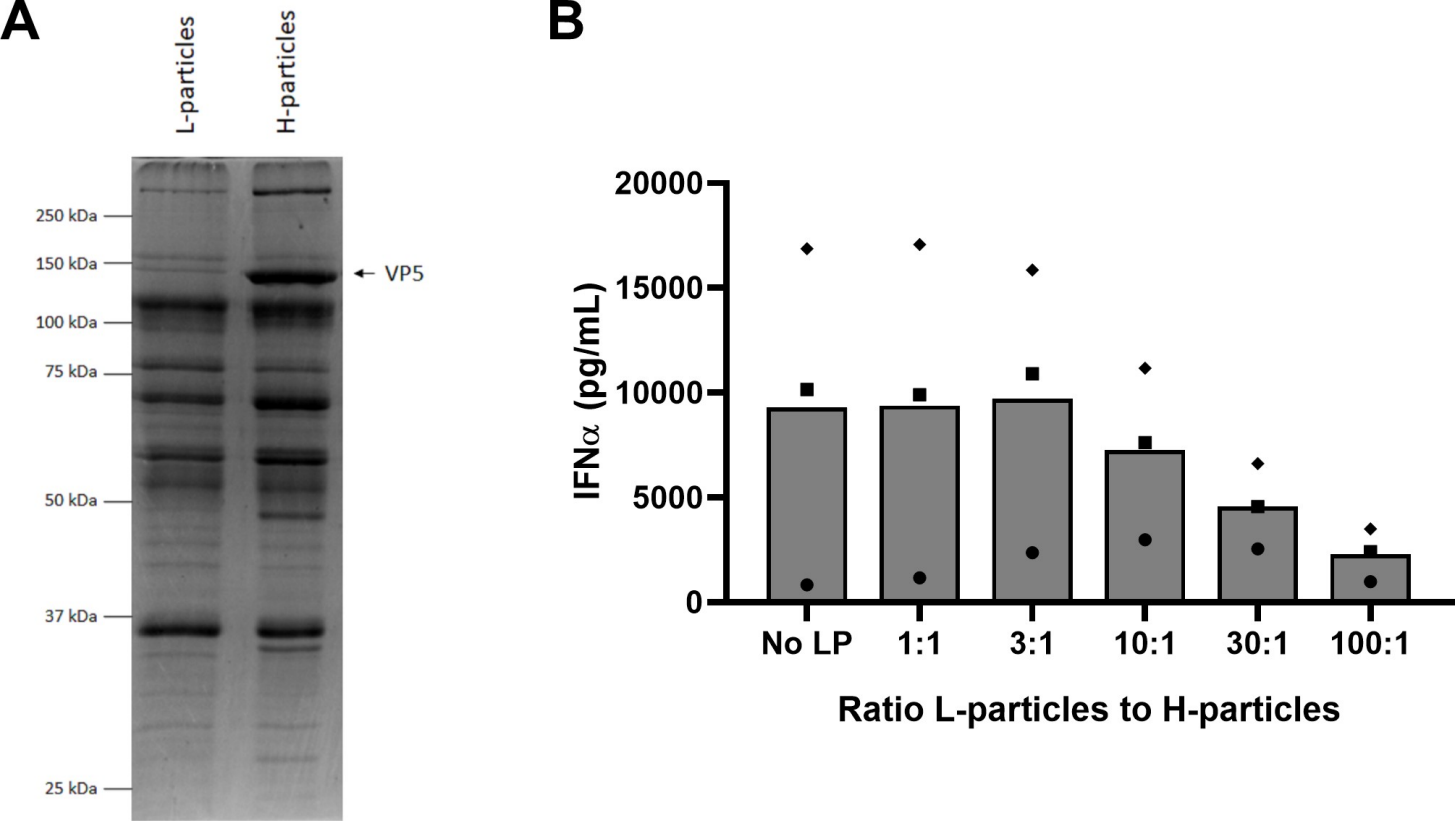
HSV-1 L-particles interfere with the H-particle-induced IFNα response by human PBMC in a dose dependent manner. (A) Coomassie blue staining of density gradient purified HSV-1 L- and H-particles. Arrow indicates position of the VP5 major capsid protein that is present in H-particles but absent from L-particles. (B) Human PBMC were incubated for 22h with density gradient purified HSV-1 H-particles and different amounts of HSV-1 L-particles. IFNα responses of PBMC of three different donors are shown.

Given that all alphaherpesviruses tested have been shown to produce L-particles [62], we explored whether the inhibitory potency of L-particles against pDC stretches beyond PRV. Therefore, HSV-1 H- and L-particles were purified by density ultracentrifugation. Although the major capsid protein VP5 was, as expected, strongly reduced in the L-article fraction as assessed by Coomassie staining (Fig 8A), it was not completely absent, suggesting a slight contamination of H-particles in the L-particle fraction, similar to what we observed for WT PRV L-particles.

Next, HSV-1 H- and L-particles were added to human primary PBMC of three different donors in similar ratios as had been tested for PRV (Fig 8B). Although a substantial variation was observed between the different donors, with one donor displaying a very weak HSV-1 H-particle-induced IFNα response by PBMC, a strong dose-dependent inhibition of the IFNα response was observed when the L-to-H-particle ratio exceeded 10, similar to what we observed for PRV. IFNα titers were not completely suppressed, although it has to be kept in mind that the HSV L-particle fraction, like the L-particle fraction of WT PRV-infected cells, is not completely devoid of H-particles, which is likely to interfere with optimal pDC inhibition by HSV-1 L-particles. In summary, we show that L-particle-mediated interference with the H-particle-induced IFNα response is a conserved feature of alphaherpesviruses.

## Discussion

**Fig 9:**
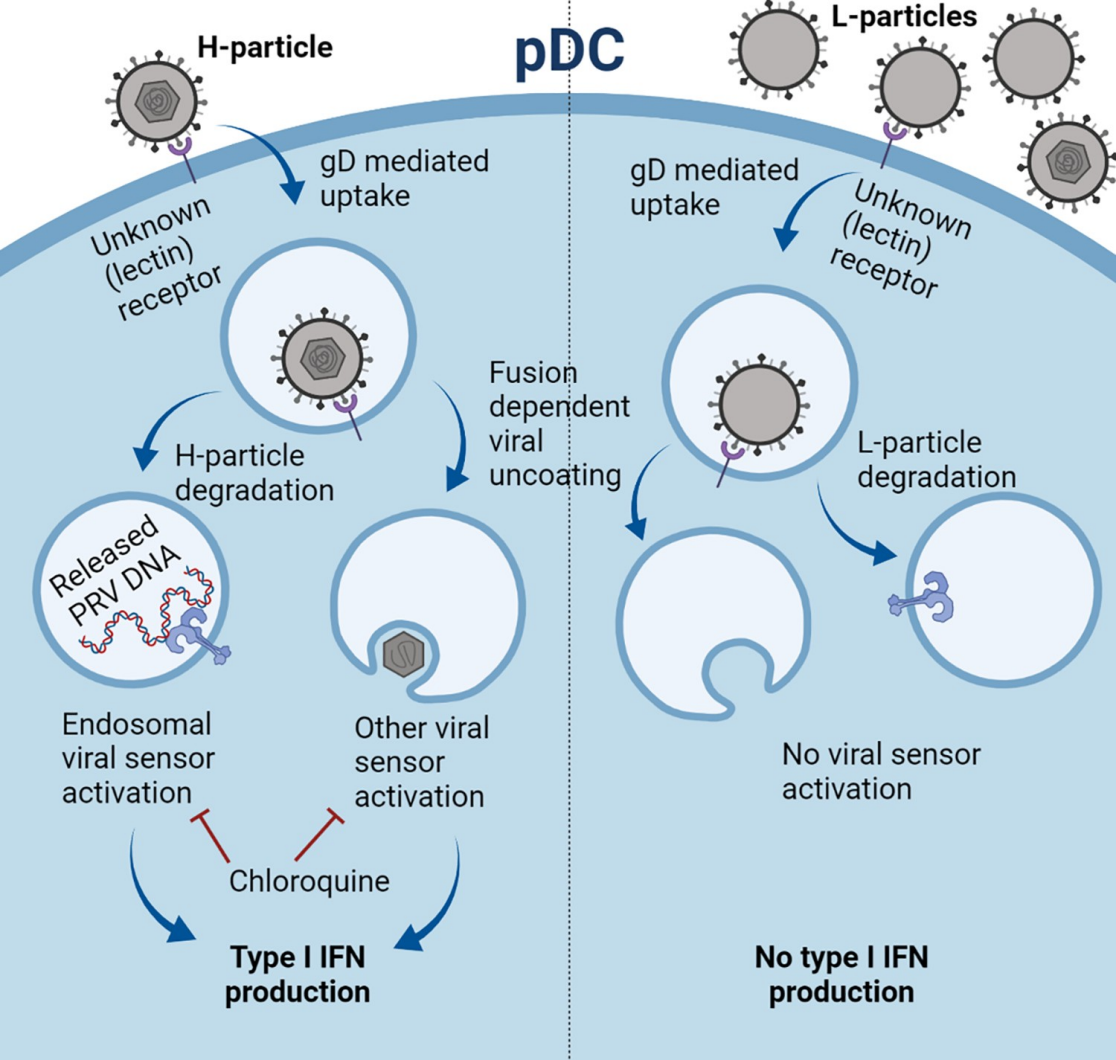
Hypothetical model of PRV H-particle induced activation and L-particle mediated inhibition of pDC. (Left) DNA-containing PRV H-particles trigger endosomal acidification- and viral gD glycoprotein-dependent activation of pDC. The viral fusion machinery contributes to but is not essential for pDC activation. (Right) DNA- and capsid-lacking viral L-particles suppress H-particle-induced activation of pDC in a competitive and gD-dependent manner. Created with BioRender.com.

In this report, we found that capsid and genomic DNA-containing H-particles are essential for an adequate induction of IFNα by PRV in primary porcine PBMC, and that this IFNα response can be virtually completely attributed to the pDC subpopulation of PBMC. We also report that efficient PRV-mediated pDC activation requires endosomal acidification and the viral gD envelope glycoprotein, and that L-particles of PRV (as well as of HSV-1) interfere with H-particle-induced IFNα production by pDC (Fig 9). This inhibition by L-particles also requires gD, which is indicative for a competitive model of inhibition and underlining the central role played by gD in the interaction of PRV with pDC. A hypothetical model on the interaction of PRV with pDC, based on the findings in the current manuscript, is shown in Fig 9.

The observation that pDC are the main source of IFNα in the PBMC population when stimulated with PRV is in line with studies on other herpesviruses, such as HSV-1 [11] or murine cytomegalovirus (MCMV) [70]. These results are not completely surprising, given that pDC can produce up to a 1,000-fold more type I IFN compared with other cell types including classical dendritic cells (cDC) or monocytes [71]. In fact, pDC have been described as the main source of systemic type I IFNs during infection of mice with several viruses [72] including MCMV and HSV-2. In addition, a marked increase in MCMV and HSV-2 viral load was observed in mice depleted for pDC [73,74]. In line with these observations in humans and mice, pDC also act as producers of large amounts of type I IFNs in response to viruses in pigs, including transmissible gastroenteritis virus (TGEV), swine influenza virus (SIV) and foot-and-mouth disease virus (FMDV) [33,75].

Type I IFNs are the most potent antiviral cytokines known in nature and the absence of pDC or type I IFNs has been shown to critically affect the outcome of virus infection, particularly alphaherpesviruses, including HSV or VZV [22,76]. Although information is scarce, the importance of the IFNα response during PRV infection has also been established. For example, in mice, type I IFNs play a crucial role in reducing the neuroinflammatory response and the clinical outcome of a PRV infection [77]. Moreover, we and others previously observed that the widely used attenuated PRV vaccine strain Bartha induces a massive IFNα response by porcine pDC *in vitro* and in mice *in vivo*, which may help to explain its reduced virulence and potent immunogenicity [32,77,78].

For HSV, it is known that genomic DNA activates pDC through the endosomal DNA-sensing TLR9 [34], in line with our current findings. To investigate whether certain PRV envelope glycoproteins are involved in pDC activation, we stimulated pDC with different PRV strains carrying deletions in particular glycoprotein-encoded genes. Mutant PRV strains that lacked one of the viral glycoproteins belonging to the gB-gH-gL fusion complex triggered a partially reduced IFN response by pDC. This suggests that, upon endosomal uptake of virions, pDC activation may not only be triggered by virus degradation and endosomal sensors including the dsDNA sensor TLR9, but may also to some extent occur upon fusion of the virus envelope with the host membrane. Future research may clarify which pDC sensors are involved in the latter, such as cytoplasmic and nuclear dsDNA sensors cGAS-STING and IFI16 that have been reported before to be triggered by herpesviruses [79,80]. Interestingly, the activation of pDC by PRV was nearly completely abolished when virions that lack the viral gD envelope glycoprotein were used, and was severely reduced by the addition of cells that stably express PRV gD, implicating that uptake of PRV virions in and subsequent activation of pDC occurs via gD. Interestingly, for HSV-1, early reports indicated that antibodies against gD, but not against gB or gC, interfere with HSV-1-induced IFNα production by PBMC [81], supporting our current data. In addition, entry-defective HSV-1 virions lacking gB and gH can induce activation of NF-κB, PI3K/Akt and Jak/Stat pathways in HFF cells, while gDnull HSV-1 virions cannot [82]. Altogether, these data demonstrate that gD functions as a multifaceted protein capable of driving specific immunological responses, including pDC activation. PRV gD has three known receptors: nectin-1 [83], nectin-2 [84] and PVR [85]. Even though we were able to confirm the expression of nectin-1 on porcine pDC, in line with what was reported for human pDC [21], cross-reactive blocking antibodies against human nectin-1 did not interfere with PRV-induced pDC activation. The nectin-1 specific CK41 antibody that was used has been confirmed to bind porcine nectin-1 and to block PRV entry in host cells [83]. Nevertheless, it cannot be entirely excluded that perhaps a lower antibody affinity for porcine nectin-1 and/or potential redundancy by other gD receptors like nectin-2 or PVR on pDC may have affected the outcome of this assay. Notwithstanding, our data suggest that a glycan-binding lectin receptor may be involved in gD-mediated activation of pDC by PRV, as removal of O- and N-linked glycans (but not of N-linked glycans alone) from PRV virions suppressed pDC activation and deglycosylated RK-13-gD cells lost their ability to suppress PRV mediated pDC activation. Many of the pDC cell surface receptors that participate in the uptake of exogenous material are lectins, including BDCA-2 [86], DCIR [87], DEC-205 [88] and Siglec-5 [89] which all recognize glycan moieties. For HIV, it was already shown that the viral glycoprotein gp120 protein binds both BDCA-2 [90] and DCIR [91]. It will be interesting to explore in future research if any and which of these lectins are involved in gD-mediated PRV-induced activation of pDC.

Although both porcine and human pDC express receptors for gD of PRV and HSV, and our current data show that gD of PRV is required for optimal IFN production by pDC, pDC do not appear to support substantial replication by either PRV (current study), HSV-1 [21] or HSV-2 [34]. Similarly, the betaherpesvirus human cytomegalovirus (HCMV) also does not productively infect human pDC [92]. It is not clear at which step of the replicative process infection is halted. Despite their ability to produce massive amounts of antiviral type I IFNs, experiments using antibody cocktails blocking type I IFN signalling illustrated that this is not the reason for the resistance of pDC against HSV or HCMV infection [21,92] and therefore unlikely the reason for the virtual lack of PRV susceptibility of porcine pDC. Both our results and the results of others show that genes under the control of an immediate early promotor are not expressed in pDC inoculated with alpha- and betaherpesviruses, indicating that the infectious cycle is interrupted at a very early stage and does not involve abortive replication, as is the case for some other viruses [75]. Interestingly and quite contrary, productive infection by the gammaherpesvirus Kaposi’s sarcoma-associated virus (KSHV) is required for pDC activation [93].

Our data also reveal that L-particles of both HSV-1 and PRV interfere with H-particle induced IFNα production by PBMC. These findings shed new light on the function of these enigmatic particles [3]. All alphaherpesviruses tested produce L-particles in cell culture, i.e. HSV-1, PRV, EHV-1, BoHV-1, VZV [56,62,94]. Our data demonstrate that the ratio of L- to H-particles is of particular importance for pDC inhibition. However, the actual ratio produced during infection differs substantially, depending on the virus and the cell type. For HSV-1, an L- to H-particle ratio of 1:1 was observed when virus was grown in BHK-21, but only 1:1,300 in Hep2 and presumably even less in Vero cells [95]. Mature monocyte derived DC cells (MoDC) on the other hand exclusively produce HSV-1 L-particles and no H-particles [8]. For BoHV-1, a little more than 1 L-particle for every H-particle is observed when the virus is grown on MDBK cells [56]. For VZV, the L-particle fraction of extracellular particles produced in MeWo cells can reach up to 85% [94]. Interestingly, when pDC were exposed to VZV-infected cells, no IFNα production could be detected [96]. Although speculative, this may point to a similar suppressive effect of VZV L-particles on pDC-mediated IFNα production as described in the current report.

L-particles lack a nucleocapsid but consist of a viral envelope enclosing the tegument and are capable of delivering their cargo into cells [97]. HSV L-particles contain a remarkably higher amount of several tegument proteins involved during the initial stages of infection [7,56,57,98]. Therefore, it has been suggested that L-particles may prepare uninfected cells for subsequent infection [97]. However, no real difference in HSV replication kinetics was observed when cells were pretreated with L-particles prior to infection [7]. Nevertheless, addition of high amounts, but not low amounts, of L-particles were able to interfere with virion adsorption [7]. These results are in line with our data showing that L-particles interfere with the effects of H-particles in a concentration-dependent manner. Heilingloh and colleagues showed that MoDC-derived L-particles of HSV-1 downregulate CD83 and the IL6 receptor on bystander MoDC [8,9], thereby suggesting immune evasive properties of L-particles. Downregulation of the IL-6 receptor was found to depend on the virion host shut-off tegument protein (pUL41) [9]. Although, theoretically, the L-particle-mediated inhibition of H-particle-induced IFN production by pDC reported in the current manuscript may be driven by L-particle-mediated delivery of inhibitory tegument protein cargo into pDC, we do not believe this is the main pathway of inhibition. Indeed, our data indicate that (i) only the IFNα response triggered by PRV H-particles and not by CpG ODN D32 is inhibited, (ii) the addition of increasing concentrations of H-particles overcomes inhibition, which correlates with a mechanism of classical competitive inhibitors [99] and (iii) removal of L-particles also removes inhibition. Combined with our data that both activation of pDC by H-particles and inhibition by L-particles depend on gD, these data add support the hypothesis that L-particles competitively interfere with H-particles for one or more pDC (gD) receptors leading to virion uptake and subsequent IFNα production. Mass spectrometry analyses have shown that the overall composition of both HSV-1 and BoHV-1 L-particles differs significantly from that of H-particles [56,98]. Of interest, HSV-1 L-particles derived from MoDC have a nearly two-fold higher content of gD than H-particles [98].

There is convincing evidence that L-particles are also produced *in vivo*. For example, vesicular fluid from HSV-1 cold sores contains particles morphologically resembling L-particles [7]. Moreover, *in vivo* PRV L-particle production was observed in epithelial cells and fibroblasts of the respiratory and olfactory mucosae of the nasal cavity of infected swine [6]. The authors also reported distinct production kinetics of H- and L-particle production *in vivo*, as they observed that cells early in infection produce many, if not exclusively, L-particles, while cells in an advanced stage of viral replication produced almost exclusively H-particles. L-particles were therefore suggested to particularly play a role during the initial stages of PRV infection in the natural host [6]. Since pDC are of major importance in rapidly producing massive amounts of IFNα early in infection [21,100], our data suggest that production of L-particles before H-particles might represent a powerful immune evasion mechanism during the initial stages of infection.

Altogether, our data reveal a role for PRV gD in the IFN response by pDC and demonstrate that L-particles interfere with H-particle-induced activation of pDC. The data therefore shed new light on alphaherpesvirus-induced type I IFN responses, which are of particular importance to keep these viruses under control.

## Supporting information

S1 Fig Blocking antibodies against nectin-1 do not affect gD-dependent pDC activation by PRV. PBMC were preincubated for 90min at 4°C with or without 10μg/mL of nectin-1-blocking antibody clone CK24 or the appropriate isotype control, followed by the addition of purified PRV, CpG or a mock control and incubated at 37°C. 22h later, the supernatant was collected and IFNα responses were measured by ELISA. Data shown are relative compared to the samples without any antibodies (set to 100) for each of two independent repeats.
(TIF)

## References

[1] Roizman B, Desrosiers RC, Fleckenstein B, Lopez C, Minson AC, Studdert MJ. The family Herpesviridae: an update. Arch Virol. 1992;123(3–4):425–49. doi: 10.1007/BF01317276 1562239

[2] Pomeranz LE, Reynolds AE, Christoph J, Hengartner CJ. Molecular Biology of Pseudorabies Virus: Impact on Neurovirology and Veterinary Medicine. Society. 2005;69(3):462–500. doi: 10.1128/MMBR.69.3.462-500.2005 16148307 PMC1197806

[3] Heilingloh CS, Krawczyk A. Role of L-particles during herpes simplex virus infection. Front Microbiol. 2017;8(2656):1–7. doi: 10.3389/fmicb.2017.02565 29312245 PMC5742154

[4] Klupp BG, Granzow H, Keil M, Mettenleiter TC. The Capsid-Associated UL25 Protein of the Alphaherpesvirus Pseudorabies Virus Is Nonessential for Cleavage and Encapsidation of Genomic DNA but Is Required for Nuclear Egress of Capsids. J Virol. 2006;80(13):6235–46. doi: 10.1128/JVI.02662-05 16775311 PMC1488961

[5] Rixon FJ, Addison C, Mclauchlan J. Assembly of enveloped tegument structures (L particles) can occur independently of virion maturation in herpes simplex virus type 1-infected cells. J Gen Virol. 1992;73(2):277–84. doi: 10.1099/0022-1317-73-2-277 1311357

[6] Aleman N, Quiroga MI, Lopez-Pena M, Vazquez S, Guerrero FH, Nieto JM. L-Particle Production during Primary Replication of Pseudorabies Virus in the Nasal Mucosa of Swine. J Virol. 2003;77(10):5657–67. doi: 10.1128/jvi.77.10.5657-5667.2003 12719558 PMC154012

[7] Dargan DJ, Subak-Sharpe JH. The effect of herpes simplex virus type 1 L-particles on virus entry, replication, and the infectivity of naked herpesvirus DNA. Virology. 1997;239(2):378–88. doi: 10.1006/viro.1997.8893 9434728

[8] Heilingloh CS, Kummer M, Mühl-Zürbes P, Drassner C, Daniel C, Klewer M, et al. L Particles Transmit Viral Proteins from Herpes Simplex Virus 1-Infected Mature Dendritic Cells to Uninfected Bystander Cells, Inducing CD83 Downmodulation. J Virol. 2015;89(21):11046–55. doi: 10.1128/JVI.01517-15 26311871 PMC4621140

[9] Birzer A, Krawczyk A, Draßner C, Kuhnt C, Mühl-Zürbes P, Heilingloh CS, et al. HSV-1 Modulates IL-6 Receptor Expression on Human Dendritic Cells. Front Immunol. 2020;11(August):1–15. doi: 10.3389/fimmu.2020.01970 32983130 PMC7479228

[10] Cella M, Jarrossay D, Facchetti F. Plasmacytoid monocytes migrate to inflamed lymph nodes and produce large amounts of type I interferon. Nat Med. 1999;5(8):919–23. doi: 10.1038/11360 10426316

[11] Siegal FP, Kadowaki N, Shodell M, Fitzgerald-Bocarsly P a, Shah K, Ho S, et al. The nature of the principal type 1 interferon-producing cells in human blood. Science. 1999;284(5421):1835–7. doi: 10.1126/science.284.5421.1835 10364556

[12] Asselin-paturel C, Boonstra A, Dalod M, Durand I, Yessaad N, Dezutter-dambuyant C, et al. Mouse type I IFN-producing cells are immature APCs with plasmacytoid morphology. Nat Immunol. 2001;2(12):1144–50. doi: 10.1038/ni736 11713464

[13] Hubert F, Voisine C, Louvet C, Heslan M, Josien R. Rat Plasmacytoid Dendritic Cells Are an Abundant Subset of MHC Class II + CD4+ CD11b−OX62− and Type I IFN-Producing Cells That Exhibit Selective Expression of Toll-Like Receptors 7 and 9 and Strong Responsiveness to CpG. J Immunol. 2004;172(12):7485–94. doi: 10.4049/jimmunol.172.12.7485 15187127

[14] Coates PTH, Barratt-boyes SM, Zhang L, Donnenberg VS, Connell PJO, Logar AJ, et al. Dendritic cell subsets in blood and lymphoid tissue of rhesus monkeys and their mobilization with Flt3 ligand. Blood J. 2003;102(7):2513–21. doi: 10.1182/blood-2002-09-2929 12829599

[15] Reid E, Juleff N, Gubbins S, Prentice H, Seago J, Charleston B, et al. Bovine Plasmacytoid Dendritic Cells Are the Major Source of Type I Interferon in Response to Foot-and-Mouth Disease Virus In Vitro and In Vivo. J Virol. 2011;85(9):4297–308. doi: 10.1128/JVI.02495-10 21307187 PMC3126242

[16] Ziegler A, Marti E, Summer A, Baumann A. Identification and characterization of equine blood plasmacytoid dendritic cells. Dev Comp Immunol. 2016;65:352–7. doi: 10.1016/j.dci.2016.08.005 27524460

[17] Guzylack-piriou L, Balmelli C, Mccullough KC. Type-A CpG oligonucleotides activate exclusively porcine natural interferon-producing cells to secrete interferon-alpha, tumour necrosis factor- alpha and interleukin-12. Immunology. 2004;112(1):28–37. doi: 10.1111/j.1365-2567.2004.01856.x 15096181 PMC1782461

[18] Colonna M, Trinchieri G, Liu YJ. Plasmacytoid dendritic cells in immunity. Nat Immunol. 2004;5(12):1219–26. doi: 10.1038/ni1141 15549123

[19] Baranek T, Zucchini N, Dalod M. Plasmacytoid dendritic cells and the control of herpesvirus infections. Viruses. 2009;1(3):383–419. doi: 10.3390/v1030383 21994554 PMC3185500

[20] Casrouge A, Zhang SY, Eidenschenk C, Jouanguy E, Puel A, Yang K, et al. Herpes simplex virus encephalitis in human UNC-93B deficiency. Science. 2006;314(5797):308–12. doi: 10.1126/science.1128346 16973841

[21] Donaghy H, Bosnjak L, Harman AN, Marsden V, Tyring SK, Meng T-C, et al. Role for plasmacytoid dendritic cells in the immune control of recurrent human herpes simplex virus infection. J Virol. 2009;83(4):1952–61. doi: 10.1128/JVI.01578-08 19073735 PMC2643779

[22] Kittan NA, Bergua A, Haupt S, Donhauser N, Schuster P, Korn K, et al. Impaired plasmacytoid dendritic cell innate immune responses in patients with herpes virus-associated acute retinal necrosis. J Immunol. 2007;179(6):4219–30. doi: 10.4049/jimmunol.179.6.4219 17785862

[23] Dalloul A, Oksenhendler E, Chosidow O, Ribaud P, Carcelain G, Louvet S, et al. Severe herpes virus (HSV-2) infection in two patients with myelodysplasia and undetectable NK cells and plasmacytoid dendritic cells in the blood. J Clin Virol. 2004;30(4):329–36. doi: 10.1016/j.jcv.2003.11.014 15163423

[24] Swiecki M, Wang Y, Gilfillan S, Colonna M. Plasmacytoid Dendritic Cells Contribute to Systemic but Not Local Antiviral Responses to HSV Infections. PLoS Pathog. 2013;9(10):2–11. 10.1371/journal.ppat.1003728 PMC3812046 24204273

[25] Swiecki M, Colonna M. Unraveling the functions of plasmacytoid dendritic cells during viral infections, autoimmunity, and tolerance. Immunol Rev. 2010;234(1):142–62. doi: 10.1111/j.0105-2896.2009.00881.x 20193017 PMC3507434

[26] Auray G, Talker SC, Keller I, Python S, Gerber M, Liniger M, et al. High-Resolution Profiling of Innate Immune Responses by Porcine Dendritic Cell Subsets in vitro and in vivo. Front Immunol. 2020;11(July):1–22. doi: 10.3389/fimmu.2020.01429 32733474 PMC7358342

[27] Villadangos A, Young L. Antigen-Presentation Properties of Plasmacytoid Dendritic Cells. Immunity. 2008;29(3):352–61. doi: 10.1016/j.immuni.2008.09.002 18799143

[28] Cella M, Facchetti F, Lanzavecchia A, Colonna M. Plasmacytoid dendritic cells activated by influenza virus and CD40L drive a potent TH1 polarization. Nat Immunol. 2000;1(4):305–10. doi: 10.1038/79747 11017101

[29] Yoneyama H, Matsuno K, Toda E, Nishiwaki T, Matsuo N, Nakano A, et al. Plasmacytoid DCs help lymph node DCs to induce anti-HSV CTLs. J Exp Med. 2005;202(3):425–35. doi: 10.1084/jem.20041961 16061729 PMC2213078

[30] Akira S, Takeda K. Toll-like receptor signalling. Nature. 2004;4(July):88–88. doi: 10.1038/nri1391 15229469

[31] Feldman SB, Ferraro M, Zheng H-M, Patel N, Gould-Fogerite S, Fitzgerald-Bocarsly P. Viral induction of Low Frequency interferon alpha producing cells. Virology. 1994;204:1–7. doi: 10.1006/viro.1994.1504 8091644

[32] Lamote JAS, Kestens M, Van Waesberghe C, Delva J, De Pelsmaeker S, Devriendt B, et al. The Pseudorabies Virus Glycoprotein gE/gI Complex Suppresses Type I Interferon Production by Plasmacytoid Dendritic Cells. J Virol. 2017;91(7):1–12. doi: 10.1128/JVI.02276-16 28122975 PMC5355608

[33] Calzada-Nova G, Schnitzlein W, Husmann R, Zuckermann F a. Characterization of the cytokine and maturation responses of pure populations of porcine plasmacytoid dendritic cells to porcine viruses and toll-like receptor agonists. Vet Immunol Immunopathol. 2010;135(1–2):20–33. doi: 10.1016/j.vetimm.2009.10.026 19939462 PMC7126865

[34] Lund J, Sato A, Akira S, Medzhitov R, Iwasaki A. Toll-like receptor 9-mediated recognition of Herpes simplex virus-2 by plasmacytoid dendritic cells. J Exp Med. 2003;198(3):513–20. doi: 10.1084/jem.20030162 12900525 PMC2194085

[35] Hochrein H, Schlatter B, O’Keeffe M, Wagner C, Schmitz F, Schiemann M, et al. Herpes simplex virus type-1 induces IFN-alpha production via Toll-like receptor 9-dependent and -independent pathways. Proc Natl Acad Sci U S A. 2004;101(31):11416–21. doi: 10.1073/pnas.0403555101 15272082 PMC509215

[36] Seeds RE, Gordon S, Miller JL. Receptors and ligands involved in viral induction of type I interferon production by plasmacytoid dendritic cells. Immunobiology. 2006;211:525–35. doi: 10.1016/j.imbio.2006.05.024 16920491 PMC7132488

[37] Nixdorf R, Klupp BG, Karger A, Mettenleiter TC. Effects of truncation of the carboxy terminus of pseudorabies virus glycoprotein B on infectivity. J Virol. 2000;74(15):7137–45. doi: 10.1128/jvi.74.15.7137-7145.2000 10888654 PMC112232

[38] Nixdorf R, Schmidt J, Karger A, Mettenleiter TC. Infection of Chinese Hamster Ovary Cells by Pseudorabies Virus. J Virol. 1999;73(10):8019–26. doi: 10.1128/JVI.73.10.8019-8026.1999 10482550 PMC112817

[39] Rauh I, Mettenleiter TC. Pseudorabies virus glycoproteins gII and gp50 are essential for virus penetration. J Virol. 1991;65(10):5348–56. doi: 10.1128/JVI.65.10.5348-5356.1991 1654444 PMC249015

[40] Babic N, Klupp BG, Makoschey B, Karger IA, Flamand A, Mettenleiter TC. Glycoprotein gH of pseudorabies virus is essential for penetration and propagation in cell culture and in the nervous system of mice. J Gen Virol. 1996;77:2277–85. doi: 10.1099/0022-1317-77-9-2277 8811028

[41] Klupp BG, Fuchs W, Weiland E. Pseudorabies Virus Glycoprotein L Is Necessary for Virus Infectivity but Dispensable for Virion Localization of Glycoprotein H. J Virol. 1997;71(10):7687–95. doi: 10.1128/JVI.71.10.7687-7695.1997 9311852 PMC192119

[42] Kaplan A, Vatter A. A comparison of herpes simplex and pseudorabies viruses. Virology. 1959;7:394–407. doi: 10.1016/0042-6822(59)90068-6 13669311

[43] Mettenleiter TC, Schreurs C, Zuckermann F, Ben-Porat T. Role of pseudorabies virus glycoprotein gI in virus release from infected cells. J Virol. 1987;61(9):2764–9. doi: 10.1128/JVI.61.9.2764-2769.1987 3039168 PMC255784

[44] Dijkstra JM, Mettenleiter TC, Klupp BG. Intracellular Processing of Pseudorabies Virus Glycoprotein M (gM): gM of Strain Bartha Lacks N-Glycosylation. Virology. 1997;122(237):113–22. doi: 10.1006/viro.1997.8766 9344913

[45] Platt K, Maré C, Hinz P. Differentiation of Vaccine Strains and Field Isolates of Pseudorabies (Aujeszky’s Disease) Virus: Thermal Sensitivity and Rabbit Virulence Markes. Arch Virol. 1979;60:13–23. doi: 10.1007/BF01318093 226030

[46] Demmin GL, Clase AC, Randall JA, Enquist LW, Banfield BW. Insertions in the gG Gene of Pseudorabies Virus Reduce Expression of the Upstream Us3 Protein and Inhibit Cell-to-Cell Spread of Virus Infection. J Virol. 2001;75(22):10856–69. doi: 10.1128/JVI.75.22.10856-10869.2001 11602726 PMC114666

[47] Smith OK. Relationship Between the Envelope and the Infectivity of Herpes Simplex Virus. Proc Soc Exp Biol Med. 1964;115:814–6. doi: 10.3181/00379727-115-29045 14155835

[48] Nauwynck HJ, Pensaert MB. Effect of specific antibodies on the cell-associated spread of pseudorabies virus in monolayers of different cell types. Arch Virol. 1995;140(6):1137–46. doi: 10.1007/BF01315422 7611884

[49] Tiels P, Verdonck F, Coddens A, Ameloot P, Goddeeris B, Cox E. Monoclonal antibodies reveal a weak interaction between the F18 fimbrial adhesin FedF and the major subunit FedA. Vet Microbiol. 2007;119(2–4):115–20. doi: 10.1016/j.vetmic.2006.08.032 17084564

[50] Van Der Stede Y, Cox E, Goddeeris BM. Antigen dose modulates the immunoglobulin isotype responses of pigs against intramuscularly administered F4-fimbriae. Vet Immunol Immunopathol. 2002;88:209–16. doi: 10.1016/s0165-2427(02)00168-x 12127418

[51] Olsen LM, Ch TH, Card JP, Enquist LW. Role of Pseudorabies Virus Us3 Protein Kinase during Neuronal Infection. J Virol. 2006;80(13):6387–98. doi: 10.1128/JVI.00352-06 16775327 PMC1488934

[52] Pescovitz M, Lunney J, Sachs D. Preparation and characterization of monoclonal antibodies reactive with porcine PBL. J Immunol. 1984;133(1):368–75. 6609988

[53] Haverson K, Bailey M, Higgins VR, Bland PW, Stokes CR. Characterization of monoclonal antibodies specific for monocytes, macrophages and granulocytes from porcine peripheral blood and mucosal tissues. J Immunol Methods. 1994;170:233–45. doi: 10.1016/0022-1759(94)90398-0 8158001

[54] Krummenacher C, Baribaud I, Leon MPDE, Whitbeck JC, Lou H, Cohen GH, et al. Localization of a Binding Site for Herpes Simplex Virus Glycoprotein D on Herpesvirus Entry Mediator C by Using Antireceptor Monoclonal Antibodies. J Virol. 2000;74(23):10863–72. doi: 10.1128/jvi.74.23.10863-10872.2000 11069980 PMC113165

[55] L’Haridon RM, Bourget P, Lefevre F, La Bonnardiere C. Production of an hybridoma library to recombinant porcine alpha I interferon: a very sensitive assay (ISBBA) allows the detection of a large number of clones. Hybridoma. 1991;10(1):35–47. doi: 10.1089/hyb.1991.10.35 2032734

[56] Russell T, Bleasdale B, Hollinshead M, Elliott G. Qualitative Differences in Capsidless L-Particles Released as a By-Product of Bovine Herpesvirus 1 and Herpes Simplex Virus 1 Infections. J Virol. 2018;92(22):1–22. doi: 10.1128/JVI.01259-18 30185590 PMC6206470

[57] Szilagyi JF, Cunningham C. Identification and characterization of a novel non-infectious herpes simplex virus-related particle. J Gen Virol. 1991;72(3):661–8. doi: 10.1099/0022-1317-72-3-661 1848601

[58] Favoreel HW, Nauwynck HJ, Halewyck HM, Van Oostveldt P, Mettenleiter TC, Pensaert MB. Antibody-induced endocytosis of viral glycoproteins and major histocompatibility complex class I on pseudorabies virus-infected monocytes. J Gen Virol. 1999;80(5):1283–91. 10355775 10.1099/0022-1317-80-5-1283

[59] Grauwet K, Cantoni C, Parodi M, De Maria A, Devriendt B, Pende D, et al. Modulation of CD112 by the alphaherpesvirus gD protein suppresses DNAM-1-dependent NK cell-mediated lysis of infected cells. Proc Natl Acad Sci U S A. 2014;111(45):16118–23. doi: 10.1073/pnas.1409485111 25352670 PMC4234607

[60] Geenen K, Favoreel HW, Olsen L, Enquist LW, Nauwynck HJ. The pseudorabies virus US3 protein kinase possesses anti-apoptotic activity that protects cells from apoptosis during infection and after treatment with sorbitol or staurosporine. Virology. 2005;331(1):144–50. doi: 10.1016/j.virol.2004.10.027 15582661

[61] Jansens RJJ, Broeck W Van Den, Pelsmaeker S De, Lamote JAS, Van Waesberghe C, Couck L, et al. Pseudorabies Virus US3-Induced Tunneling Nanotubes Contain Stabilized Microtubules, Interact with Neighboring Cells via Cadherins, and Allow Intercellular Molecular Communication. J Virol. 2017;91(19):1–13. 10.1128/JVI.00749-17 PMC5599745 28747498

[62] Mclauchlan J, Rixon FJ. Characterization of enveloped tegument structures (L particles) produced by alphaherpesviruses: integrity of the tegument does not depend on the presence of capsid or envelope. J Gen Virol. 1992;73:269–76. doi: 10.1099/0022-1317-73-2-269 1311356

[63] Ankel H, Westra DF, Welling-wester S, Lebon P, Rene Â. Induction of Interferon-alpha by Glycoprotein D of Herpes Simplex Virus: A Possible Role of Chemokine Receptors. Virology. 1998;251:317–26. doi: 10.1006/viro.1998.9432 9837796

[64] Lee HK, Lund JM, Ramanathan B, Mizushima N, Iwasaki A. Autophagy-dependent viral recognition by plasmacytoid dendritic cells. Science. 2007;315(5817):1398–401. doi: 10.1126/science.1136880 17272685

[65] KLEYMANN, GERALD FISCHER R, BETZ U, HENDRIX M, BENDER W, SCHNEIDER U, HANDKE G, et al. New helicase-primase inhibitors as drug candidates for the treatment of herpes simplex disease. Nat Med. 2002;8(4):392–8. doi: 10.1038/nm0402-392 11927946

[66] Vallbracht M, Backovic M, Klupp BG, Rey FA, Mettenleiter TC. Common characteristics and unique features: A comparison of the fusion machinery of the alphaherpesviruses Pseudorabies virus and Herpes simplex virus. 1st ed. Vol. 104, Advances in Virus Research. Elsevier Inc.; 2019. 225–281 p. 10.1016/bs.aivir.2019.05.007 31439150

[67] Reske A, Pollara G, Krummenacher C, Katz R, Chain BM. Glycoprotein-Dependent and TLR2-Independent Innate Immune Recognition of Herpes Simplex Virus-1 by Dendritic Cells. J Immunol. 2008;180:7525–36. doi: 10.4049/jimmunol.180.11.7525 18490753

[68] Spear PG. Entry of alphaherpesviruses into cells. Vol. 4, Seminars in Virology. 1993. p. 167–80.

[69] Petrovskis EA, Timmins JG, Armentrout MA, Marchioli CC, Yancey RJ, Post LE. DNA Sequence of the Gene for Pseudorabies Virus gp50, a Glycoprotein without N-Linked Glycosylation. J Virol. 1986;59(2):216–23. doi: 10.1128/JVI.59.2.216-223.1986 3016293 PMC253069

[70] Zucchini N, Bessou G, Robbins SH, Chasson L, Raper A, Crocker PR, et al. Individual plasmacytoid dendritic cells are major contributors to the production of multiple innate cytokines in an organ-specific manner during viral infection. Int Immunol. 2007;20(1):45–56. doi: 10.1093/intimm/dxm119 18000008 PMC7110020

[71] Ito T, Kanzler H, Duramad O, Cao W, Liu YJ. Specialization, kinetics, and repertoire of type 1 interferon responses by human plasmacytoid predendritic cells. Blood. 2006;107(6):2423–31. doi: 10.1182/blood-2005-07-2709 16293610

[72] Ali S, Mann-nüttel R, Schulze A, Richter L, Alferink J. Sources of Type I Interferons in Infectious Immunity: Plasmacytoid Dendritic Cells Not Always in the Driver’ s Seat. Front Immunol. 2019;10(778):1–20. doi: 10.3389/fimmu.2019.00778 31031767 PMC6473462

[73] Dalod M, Salazar-mather TP, Malmgaard L, Lewis C, Asselin-paturel C, Brière F, et al. Interferon alpha/beta and Interleukin 12 Responses to Viral Infections: Pathways Regulating Dendritic Cell Cytokine Expression In Vivo. J Exp Med. 2002;195(4):517–28. doi: 10.1084/jem.20011672 11854364 PMC2193614

[74] Stout-delgado HW, Yang X, Walker WE, Tesar BM, Goldstein DR. Aging Impairs IFN Regulatory Factor 7 Up-Regulation in Plasmacytoid Dendritic Cells during TLR9 Activation. J Immunol. 2008;181:6747–56. doi: 10.4049/jimmunol.181.10.6747 18981092 PMC2605669

[75] Guzylack-Piriou L, Bergamin F, Gerber M, McCullough KC, Summerfield A. Plasmacytoid dendritic cell activation by foot-and-mouth disease virus requires immune complexes. Eur J Immunol. 2006;36(7):1674–83. doi: 10.1002/eji.200635866 16783856

[76] Jamali A, Hu K, Sendra VG, Blanco T, Lopez MJ, Ortiz G, et al. Characterization of Resident Corneal Plasmacytoid Dendritic Cells and Their Pivotal Role in Herpes Simplex Keratitis. Cell Rep. 2020;32(9):1–17. doi: 10.1016/j.celrep.2020.108099 32877681 PMC7511260

[77] Laval K, Van Cleemput J, Vernejoul JB, Enquist LW. Alphaherpesvirus infection of mice primes PNS neurons to an inflammatory state regulated by TLR2 and type i IFN signaling. PLoS Pathog. 2019;15(11):1–21. 10.1371/journal.ppat.1008087 PMC6824567 31675371

[78] Delva JL, Nauwynck HJ, Mettenleiter TC, Favoreel HW. The Attenuated Pseudorabies Virus Vaccine Strain Bartha K61: A Brief Review on the Knowledge Gathered During 60 Years of Research. Pathogens. 2020;9:1–13. doi: 10.3390/pathogens9110897 33121171 PMC7693725

[79] Diner BA, Lum KK, Javitt A, Cristea IM. Interactions of the antiviral factor IFI16 mediate immune signaling and herpes simplex virus-1 immunosuppression. Mol Cell proteomics. 2015;14(9):2341–56. doi: 10.1074/mcp.M114.047068 25693804 PMC4563720

[80] Zheng C. A Tug of War: DNA-Sensing Antiviral Innate Immunity and Herpes Simplex Virus Type I Infection. Front Immunol. 2019;10(2627):1–9. doi: 10.3389/fmicb.2019.02627 31849849 PMC6901958

[81] Lebon P. Inhibition of Herpes Simplex Virus Type 1-induced Interferon Synthesis by Monoclonal Antibodies against Viral Glycoprotein D and by Lysosomotropic Drugs. J Gen Virol. 1985;66:2781–6. doi: 10.1099/0022-1317-66-12-2781 2999320

[82] Macleod IJ, Minson T. Binding of Herpes Simplex Virus Type-1 Virions Leads to the Induction of Intracellular Signalling in the Absence of Virus Entry. PLoS One. 2010;5(3):1–12. doi: 10.1371/journal.pone.0009560 20221426 PMC2832691

[83] Milne RSB, Connolly SA, Krummenacher C, Eisenberg RJ, Cohen GH. Porcine HveC, a member of the highly conserved HveC/nectin 1 family, is a functional alphaherpesvirus receptor. Virology. 2001;281(2):315–28. doi: 10.1006/viro.2000.0798 11277703

[84] Warner MS, Geraghty RJ, Martinez WM, Montgomery RI, Whitbeck JC, Xu R, et al. A cell surface protein with herpesvirus entry activity (Hveb) confers susceptibility to infection by mutants of herpes simplex virus type 1, herpes simplex virus type 2, and pseudorabies virus. Virology. 1998;246(1):179–89. doi: 10.1006/viro.1998.9218 9657005

[85] Geraghty RJ, Krummenacher C, Cohen GH, Eisenberg RJ, Spear PG. Entry of alphaherpesviruses mediated by poliovirus receptor-related protein 1 and poliovirus receptor. Science. 1998;280(5369):1618–20. doi: 10.1126/science.280.5369.1618 9616127

[86] Jaehn PS, Zaenker KS, Schmitz J, Dzionek A. Functional dichotomy of plasmacytoid dendritic cells: Antigen-specific activation of T cells versus production of type I interferon. Eur J Immunol. 2008;38(7):1822–32. doi: 10.1002/eji.200737552 18581320

[87] Meyer-wentrup F, Benitez-ribas D, Tacken PJ, Punt CJ a Figdor CG, Vries IJM De, et al. Targeting DCIR on human plasmacytoid dendritic cells results in antigen presentation and inhibits IFN-a production. Blood. 2008;111(8):4245–53. doi: 10.1182/blood-2007-03-081398 18258799

[88] Tel J, Benitez-Ribas D, Hoosemans S, Cambi A, Adema GJ, Figdor CG, et al. DEC-205 mediates antigen uptake and presentation by both resting and activated human plasmacytoid dendritic cells. Eur J Immunol. 2011;41(4):1014–23. doi: 10.1002/eji.201040790 21413003

[89] Escalona Z, Álvarez B, Uenishi H, Toki D, Yuste M, Revilla C, et al. Molecular characterization and expression of porcine Siglec-5. Dev Comp Immunol. 2014;44:206–16. doi: 10.1016/j.dci.2013.12.013 24382335

[90] Martinelli E, Cicala C, Van Ryk D, Goode DJ, Macleod K, Arthos J, et al. HIV-1 gp120 inhibits TLR9-mediated activation and IFN-alpha secretion in plasmacytoid dendritic cells. Proc Natl Acad Sci U S A. 2007;104(9):3396–401. doi: 10.1073/pnas.0611353104 17360657 PMC1805537

[91] Jin W, Li C, Du T, Hu K, Huang X, Hu Q. DC-SIGN plays a stronger role than DCIR in mediating HIV-1 capture and transfer. Virology. 2014;458–459(1):83–92. 10.1016/j.virol.2014.04.016 24928041

[92] Kvale EØ, Dalgaard J, Lund-johansen F, Rollag H, Farkas L, Midtvedt K, et al. CD11c+ dendritic cells and plasmacytoid DCs are activated by human cytomegalovirus and retain efficient T cell–stimulatory capability upon infection. Blood J. 2022;107(5):2022–9. 10.1182/blood-2005-05-2016 16269620

[93] West JA, Gregory SM, Sivaraman V, Su L, Damania B. Activation of Plasmacytoid Dendritic Cells by Kaposi’s Sarcome-Associated Herpesvirus. J Virol. 2011;85(2):895–904. doi: 10.1128/JVI.01007-10 20980519 PMC3020034

[94] Carpenter JE, Hutchinson JA, Jackson W, Grose C. Egress of Light Particles among Filopodia on the Surface of Varicella-Zoster Virus-Infected Cells. J Virol. 2008;82(6):2821–35. doi: 10.1128/JVI.01821-07 18184710 PMC2258984

[95] Yang Y-T, Courtney R. Influence of the Host Cell on the Association of ICP4 and ICP0 with Herpes Simplex Virus Type 1. Virology. 1995;211:209–17. doi: 10.1006/viro.1995.1393 7645212

[96] Huch JH, Cunningham L, Arvin M, Nasr N, Santegoets SJ a. M, Slobedman E, et al. Impact of Varicella-Zoster Virus on Dendritic Cell Subsets in Human Skin during Natural Infection. J Virol. 2010;84(8):4060–72. doi: 10.1128/JVI.01450-09 20130046 PMC2849518

[97] McLauchlan J, Addison C, Craigie MC, Rixon FJ. Noninfectious L-particles supply functions which can facilitate infection by HSV-1. Virology. 1992;190(2):682–8. doi: 10.1016/0042-6822(92)90906-6 1325700

[98] Birzer A, Kraner, Max Edmund Heilingloh CS, Mühl-Zürbes P, Hofmann J, Steinkasserer A, Popella L. Mass Spectrometric Characterization of HSV-1 L-Particles From Human Dendritic Cells and BHK21 Cells and Analysis of Their Functional Role. Front Microbiol. 2020;11(1997):1–24. doi: 10.3389/fmicb.2020.01997 33117298 PMC7550753

[99] Kenakin T. Enzymes as Drug Targets. In: Pharmacology in Drug Discovery and Development. 2017. p. 131–56.

[100] Lund JM, Linehan MM, Iijima N, Iwasaki A. Cutting Edge: Plasmacytoid Dendritic Cells Provide Innate Immune Protection against Mucosal Viral Infection In Situ. J Immunol. 2006;177(11):7510–4. doi: 10.4049/jimmunol.177.11.7510 17114418

