# Supplementary figures and images for: Alphaherpesvirus-induced activation of plasmacytoid dendritic cells depends on the viral glycoprotein gD and is inhibited by non-infectious light particles
Source: PLoS Pathog. 2021 Nov 29;17(11):e1010117. doi: 10.1371/journal.ppat.1010117 (PMC8659615; doi:10.1371/journal.ppat.1010117)

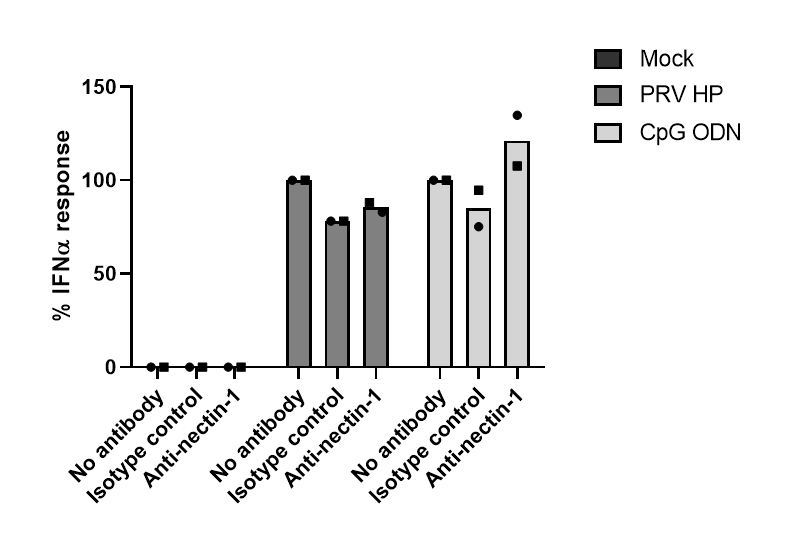

Supplement: S1 Fig — PBMC were preincubated for 90min at 4°C with or without 10μg/mL of nectin-1-blocking antibody clone CK24 or the appropriate isotype control, followed by the addition of purified PRV, CpG or a mock control and incubated at 37°C. 22h later, the supernatant was collected and IFNα responses were measured by ELISA. Data shown are relative compared to the samples without any antibodies (set to 100) for each of two independent repeats. (TIF) [file ppat.1010117.s001.tif]
